# Population Pharmacokinetics and Extrapolation of NP-011 from Animal to Humans: A Novel Therapeutic Candidate Protein for Metabolic Dysfunction-Associated Steatohepatitis

**DOI:** 10.3390/pharmaceutics18091194

**Published:** 2026-09-21

**Authors:** Hyunjung Lee, Quyen Thi Tran, Bui Thi Tham, Min-Kyoung Kim, Hwi-Yeol Yun, Jung-Woo Chae, Jung-Hyuck Park, Soyoung Lee

**Affiliations:** 1 College of Pharmacy, Chungnam National University, Daejeon 34134, Republic of Korea; hjung0222@gmail.com (H.L.); hyyun@cnu.ac.kr (H.-Y.Y.); jwchae@cnu.ac.kr (J.-W.C.); 2 Department of Bio-AI Convergence, Chungnam National University, Daejeon 34134, Republic of Korea; 3 Faculty of Pharmacy, PHENIKAA University, Nguyen Trac, Duong Noi, Hanoi 12116, Vietnam; quyen.tranthi@phenikaa-uni.edu.vn; 4 Faculty of Pharmacy, Haiphong University of Medicine and Pharmacy, Haiphong 18000, Vietnam; bttham@hpmu.edu.vn; 5 NEXEL Co., Ltd., Seoul 07802, Republic of Korea; sweva100@naver.com; 6 Bio-AI Convergence Research Center, Chungnam National University, Daejeon 34134, Republic of Korea; 7 QuBEST BIO Co., Ltd., Yongin 17015, Republic of Korea

**Keywords:** NP-011, population pharmacokinetics, allometric scaling, pharmacokinetic prediction, MASH

## Abstract

**Background/Objective:** Metabolic dysfunction-associated steatohepatitis (MASH), an advanced stage of metabolic dysfunction-associated steatotic liver disease (MASLD), is characterized by progressive liver fibrosis and represents an important therapeutic target. NP-011 is a truncated recombinant human milk fat globule EGF factor 8 protein developed for the treatment of MASH-associated liver fibrosis. This study extrapolated the pharmacokinetics of NP-011 from animals to humans and retrospectively evaluated the predictions using Phase I clinical data. **Methods:** Population pharmacokinetic models were developed using plasma concentration–time data following the intravenous administration of NP-011 in mice, rats, and monkeys. Model performance was evaluated using goodness-of-fit diagnostics, visual predictive checks, and nonparametric bootstrap analyses. The human pharmacokinetic parameters were predicted using simple allometric scaling. The maximum lifespan potential-corrected scaling was evaluated as a supplementary sensitivity analysis. Predicted human concentration–time profiles and exposure parameters were retrospectively compared with observed data from the single ascending dose component of the Phase I data. The Phase I study was registered at ClinicalTrials.gov (NCT05387499). **Results:** A two-compartment model with first-order elimination described NP-011 pharmacokinetics in all three species. For a 70-kg adult, simple allometry predicted clearance, central volume of distribution, intercompartmental clearance, and peripheral volume of distribution values of 3.80 L/h, 2.88 L, 0.25 L/h and 24.61 L, respectively. Across the 0.25–4 mg dose range, the predicted median AUC_inf_ values were 1.62- to 1.96-fold higher than the corresponding observed medians, whereas the predicted median C_max_ values were 1.33- to 1.48-fold higher. Overall 17.1% of the observed concentrations were contained within the simulated 5th–95th percentile prediction intervals. The maximum lifespan potential correction resulted in greater overprediction of AUC_inf_, with predicted-to-observed ratios of 2.38–2.89, whereas C_max_ predictions were unchanged. **Conclusions:** Simple allometry provided closer agreement with the observed Phase I exposure than maximum lifespan potential-corrected scaling. However, the systematic overprediction of exposure and limited coverage of the simulated prediction intervals indicate that these predictions should be interpreted cautiously. These findings support simple allometry as a practical approach for extrapolating the pharmacokinetics of NP-011 to healthy adults in early clinical development, although broader generalization requires further evaluation.

## 1. Introduction

Metabolic dysfunction-associated steatohepatitis (MASH), formerly termed non-alcoholic steatohepatitis, is an advanced form of metabolic dysfunction-associated steatotic liver disease (MASLD, formerly non-alcoholic fatty liver disease). MASH is characterized histologically by hepatic steatosis, hepatocellular ballooning, and lobular inflammation and may progress to fibrosis, cirrhosis, and hepatocellular carcinoma [1,2,3,4]. Given the increasing prevalence and substantial clinical burden of MASLD and MASH, the development of effective anti-fibrotic therapies is an important and unmet medical need.

Multiple therapeutic strategies targeting metabolic dysfunctions, inflammation, and fibrosis have been investigated for MASH [5,6,7,8]. The heterogeneous pathophysiology of MASH and the limited availability of effective antifibrotic treatments continue to present challenges in drug development.

NP-011 is a novel protein therapeutic candidate developed by NEXEL Co. Ltd. for the treatment of MASH-associated liver fibrosis. NP-011 is a truncated, recombinant, human milk fat globule-EGF factor 8 protein that binds integrin αvβ5 with a reported equilibrium dissociation constant of 2.0 nM and inhibits the interaction between integrin αvβ5 and the transforming growth factor-β (TGF- β) receptor [9]. NP-011 is a truncated MFG-E8 protein with an apparent molecular mass of 20–30 kDa under reducing conditions and approximately 20 kDa under non-reducing conditions, as determined by SDS-PAGE. This activity attenuates TGF-β signaling, hepatic stellate cell activation, and fibrogenesis. Characterization of the pharmacokinetics (PK) of NP-011 and translation of preclinical findings to humans are therefore important for supporting its clinical development.

Preclinical studies and Phase I trials are essential for the discovery and development of new compounds. Preclinical trials involve in vitro and in vivo studies to gather data for guiding future clinical research. The goal is to ensure that only the safest and most promising candidates advance to human testing. Animal studies are crucial for evaluating PK and simulating human disease conditions and physiological responses, providing essential insights into potential treatment effects.

Interspecies PK extrapolation is frequently used during early drug development to support first-in-human dose selection when clinical PK data are unavailable. Allometric scaling relates PK parameters to body size across species and has been widely applied for the prediction of human clearance and distribution volumes. However, the predictive performance may depend on the species included, the estimated scaling exponent, and the use of correction factors. For therapeutic proteins, allometric scaling has also been used to extrapolate clearance and distribution parameters from preclinical species to humans, with simple allometry and exponent-based correction approaches applied depending on the parameter and scaling relationship. Therefore, the evaluation of extrapolated predictions against subsequently obtained clinical data is important to determine the suitability of a given scaling approach.

This study aimed to develop population PK models of NP-011 using plasma concentration–time data from mice, rats, and monkeys; extrapolate the model parameters to humans using interspecies allometric scaling; and retrospectively evaluate the predicted human PK in healthy adults using the observed phase I data.

**Scheme 1:**
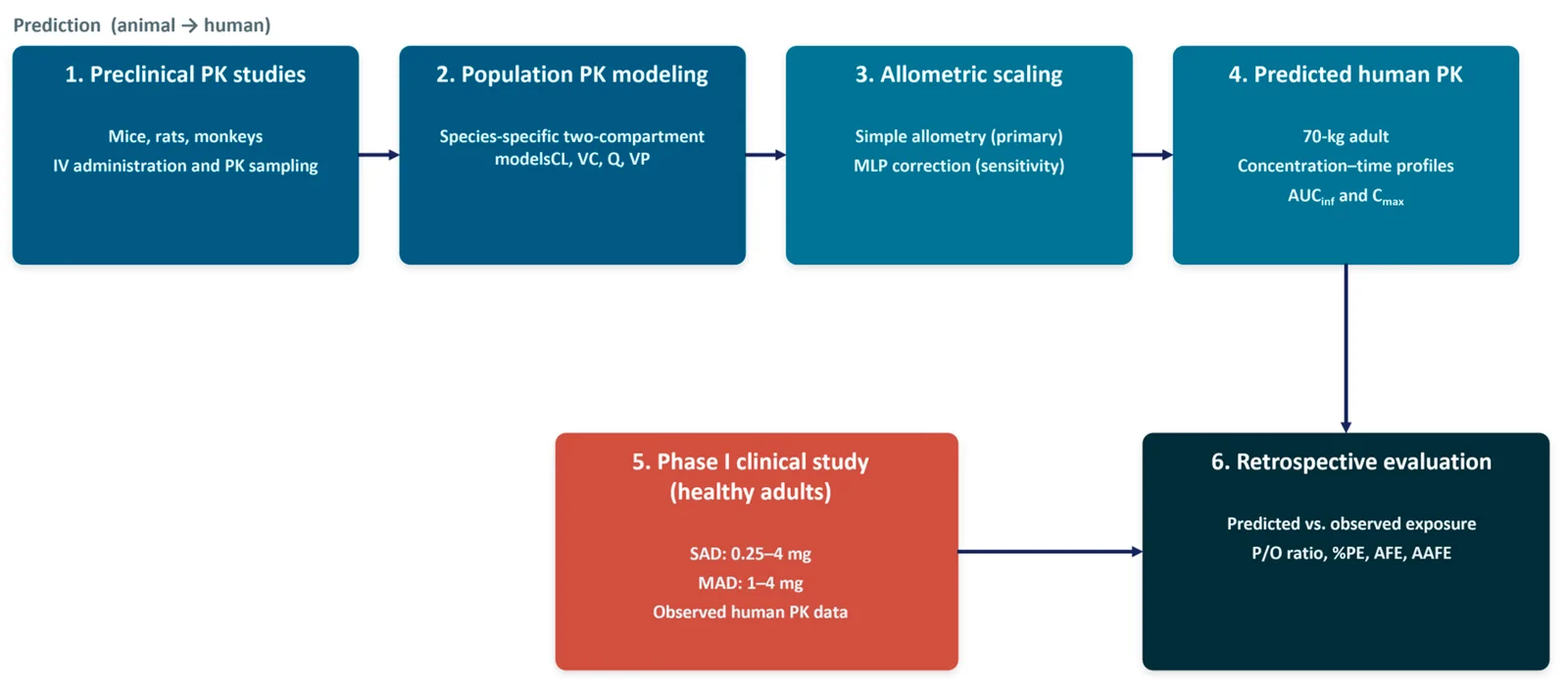
Overall workflow of NP-011 pharmacokinetic modeling and human extrapolation.

The overall study workflow is summarized in Scheme 1.

## 2. Materials and Methods

### 2.1. Study Design

#### 2.1.1. Preclinical Studies

The PK profiles of NP-011 after single or repeated intravenous (IV) bolus administration were evaluated in mice, rats, and monkeys. In repeated-dose studies, plasma samples were collected before dosing and at 0.083, 0.5, 1, 2, 4, 6, 8, and 24 h after administration on the first and last dosing days (days 1 and 14, respectively). Additional predose samples were collected once daily from days 3–13. Plasma NP-011 concentrations were quantified using enzyme-linked immunosorbent assay (ELISA). The ELISA method used for the preclinical studies was validated with respect to calibration and quantification ranges, accuracy, precision, dilutional linearity, hook effect, and stability according to predefined acceptance criteria. PK parameters were derived by non-compartmental analysis (NCA) using Phoenix WinNonlin 7.0. Detailed study designs, including animal species, dose levels, number of animals, dosing regimens, and sampling schedules, are provided in Table S1.

#### 2.1.2. Phase I Trials

A Phase I, randomized, double-blind, placebo-controlled study was conducted to evaluate the safety, tolerability, and PK of NP-011, a truncated recombinant human MFG-E8 protein, following IV bolus administration to healthy volunteers aged 18–65 years. The trial was registered at ClinicalTrials.gov (NCT05387499; https://clinicaltrials.gov/study/NCT05387499, accessed on 11 August 2026).

A total of 66 participants were randomized and dosed across the two study parts. Part 1 was a single ascending dose (SAD) study comprising 40 participants across five dose cohorts (0.25, 0.5, 1, 2, and 4 mg). In each cohort, six participants received NP-011 and two received placebo and completed the SAD part, and all 30 NP-011-treated participants were included in the PK population. Part 2 was a multiple ascending dose (MAD) study comprising 26 participants across three dose cohorts (1, 2, and 4 mg), including 19 participants who received NP-011 and seven who received placebo. Study treatment was administered once daily for seven consecutive days. One NP-011-treated participant and one placebo-treated participant discontinued after the first dose because of poor venous access; however, all 19 participants who received NP-011 were included in the PK population. Detailed cohort allocations, participant disposition, and PK sampling schedules are provided in Table S2.

Serial plasma samples were collected to characterize the PK of NP-011. For the SAD part, PK samples were obtained predose and at 0.083, 0.25, 0.5, 1, 2, 4, 8, 12, 24, and 48 h after dosing. Plasma NP-011 concentrations were quantified using a validated ELISA in accordance with the clinical trial protocol, with an LLOQ of 15.62 ng/mL. Anti-drug antibodies (ADA), including neutralizing antibodies, were also assessed according to the clinical study protocol. Safety was assessed through adverse event monitoring, and the study was conducted in accordance with the International Council for Harmonization Good Clinical Practice (ICH-GCP) guidelines and applicable ethical and regulatory requirements.

The present manuscript represents secondary pharmacokinetic modeling and retrospective evaluation of data from the Phase I randomized trial rather than a primary report of the randomized trial. Accordingly, CONSORT 2025 was used as a reporting reference for applicable trial-related information. A completed CONSORT 2025 checklist and participant-flow diagrams for the SAD and MAD parts are provided as separate files accompanying this submission.

### 2.2. Model Development

#### 2.2.1. Non-Compartment Analysis (NCA)

Preclinical plasma concentration–time data were analyzed separately for each animal species and dose group using NCA. The area under the plasma concentration–time curve was calculated using the linear-up/log-down trapezoidal method, and PK parameters including C_max_, AUC_last_, AUC_inf_, terminal half-life (*t*_1/2_), total drug clearance (CL), volume of distribution (*V*_d_), and initial concentration (*C*_0_) were determined. For NCA, BLQ concentrations at predose and before T_max_ were set to zero, whereas BLQ observations after T_max_ were treated as missing or excluded from the analysis. The detailed calculation methods and specific results are provided in Tables S3–S5.

#### 2.2.2. Population PK Model Development and Evaluation

Population PK models were developed separately for mice, rats, and monkeys using nonlinear mixed-effects modeling in NONMEM (version 7.5.0) to characterize the plasma concentration–time profile of NP-011 following IV administration. One- and two-compartment structural models with first-order elimination were also evaluated. BLQ observations were excluded from the population PK modeling datasets using an M1 approach; likelihood-based M3 or M4 methods were not applied.

Inter-individual variability in the PK parameters was described using an exponential model, with the individual random effect assumed to follow a normal distribution with a mean of 0 and a variance of $\omega^{2}$. The additive, proportional, and combined additive and proportional residual models were evaluated.

Model selection was based on changes in the objective function value (OFV), parameter precision, and goodness-of-fit (GOF) diagnostic plots, including observed versus predicted concentrations and residual plots. For nested models, statistical significance was assessed using the likelihood-ratio test, with the degrees of freedom corresponding to the difference in the number of estimated parameters and the corresponding chi-square critical value at α = 0.05. The predictive performance was further assessed using visual predictive checks by comparing the observed concentrations with the simulated median and the 5th and 95th percentiles. Final model adequacy was additionally evaluated using ETA distributions and shrinkage and parameter-correlation matrices.

The robustness and precision of the final parameter estimates were assessed using a nonparametric bootstrap analysis based on 1000 resampled datasets. Individual animals rather than individual records were resampled, thereby preserving the complete within-animal observation sequence in each bootstrap replicate. The median parameter estimates and the corresponding 95% confidence intervals obtained from the bootstrap distributions were compared with the final model estimates. Bootstrap success rates, near-boundary estimates, and failure modes were also evaluated.

### 2.3. Allometric Scaling

Human PK parameters were extrapolated from the final population PK estimates obtained in mice, rats, and monkeys using simple allometric scaling (SA). Clearance (CL), central volume of distribution (*V_C_*), intercompartmental clearance (*Q*), and peripheral volume of distribution (*V_P_*) were scaled according to the following power function:

(1) $$Y=a*{BW}^{b}$$

where *Y* represents the PK parameter of interest, BW represents body weight, and a and b represent the allometric coefficient and exponent, respectively. The body weight-normalized parameters reported in Table 1 were converted to total species-level parameters by multiplying each normalized estimate by the corresponding species-specific body weight before allometric regression. The allometric relationship described in Equation (1) was fitted by ordinary least-squares regression using log-transformed total parameter values and body weights. The regression was unweighted, and residuals were assumed to be independent with constant variance on the log scale. Human values were obtained by extrapolation to a reference body weight of 70 kg. Sensitivity of the fitted allometric exponents to the individual source species was assessed using leave-one-species-out analyses, in which the regression was repeated after sequential exclusion of mouse, rat, or monkey data.

For human PK prediction, the inter-individual variability was assumed to be the same as that estimated from the monkey population PK model. Residual variability was not incorporated into the human simulation intervals. In addition to SA, the rule of exponents (ROE) was considered for CL and *Q* to determine whether an additional correction factor was required based on the estimated allometric exponent [10,11]. According to the ROE criteria summarized in Table S8, maximum lifespan potential (MLP) correction was applied when the estimated exponent fell within the range of 0.7 ≤ *b* ≤ 1.0. Detailed equations and procedures for the MLP-based scaling approach are provided in the Supplementary Information.

### 2.4. Retrospective Evaluation Using Human PK Profiles

Human PK parameters were not estimated from the clinical data; rather, they were obtained by simple allometric scaling of the animal population PK estimates and fixed in the model used for simulation. The Phase I clinical data were used solely for retrospective evaluation of these predictions. The quantitative retrospective evaluation was restricted to the SAD component of the Phase I study; the MAD concentration–time profiles were not included in the formal comparison of predicted and observed PK.

The human PK profiles of NP-011 were predicted by incorporating human CL, *V_C_*, *Q*, and *V_P_* values derived using simple allometric scaling into the final population PK model. Simulations were conducted for each dose level evaluated in the Phase I clinical trial, and the simulated median concentration–time profiles and the corresponding 5th and 95th percentiles were generated.

For the human simulations, NP-011 was implemented as an instantaneous intravenous bolus into the central compartment (CMT = 1), with dose amounts entered in micrograms (250, 500, 1000, 2000, and 4000 μg, respectively). No bioavailability terms were specified. Bioavailability was therefore unity by default, consistent with intravenous administration. The model was implemented using ADVAN6 with user-defined differential equations. The central plasma concentration was defined as the amount in the central compartment divided by *V_C_*, and C_max_ was obtained as the maximum simulated central-compartment concentration. For direct comparison with the observed Phase I AUC_inf_, the predicted AUC_inf_ was calculated for each simulation replicate as Dose/CL.

Predictive performance was retrospectively evaluated by overlaying the observed Phase I plasma concentrations on the simulated median profiles and their 5th–95th percentile ranges. Comparisons of AUC_inf_ and *C*_max_ were performed using dose-group summary values, with the observed cohort medians compared with the corresponding median predictions. The predicted-to-observed ratio (P/O), percentage prediction error (%PE), average fold error (AFE), and absolute average fold error (AAFE) were calculated as follows:

(2) $$P/O=\frac{P_{i}}{O_{i}}$$

(3) $$\%PE=\frac{P_{i}-O_{i}}{O_{i}}\times 100$$

(4) $$AFE=10^{\frac{1}{n}\sum_{i=1}^{n}{log}_{10}\left(\frac{P_{i}}{O_{i}}\right)}$$

(5) $$AAFE=10^{\frac{1}{n}\sum_{i=1}^{n}\left|{log}_{10}\left(\frac{P_{i}}{O_{i}}\right)\right|}$$

where *P_i_* and *O_i_* denote the predicted and observed dose-group median values, respectively. AFE was used as a measure of bias, whereas AAFE was used as a measure of precision. Coverage of the simulated 5th–95th percentile interval was quantified as the number and proportion of observed concentrations falling within the interval at each dose level and overall.

For the two-compartment model, the micro-rate constants were calculated as *k*_10_ = *CL*/*V_C_*, *k*_12_ = *Q/V_C_*, and *k*_21_ = *Q/V_P_*. The hybrid disposition exponents (α and β) were then obtained from

(6) $$\alpha ,\beta =\frac{\left(k_{10}+k_{12}+k_{21}\right)\;\pm \;\sqrt{{\left(k_{10}+k_{12}+k_{21}\right)}^{2}-{4k}_{10}k_{21}}}{2}$$

with α > β. The distribution and terminal half-lives were subsequently derived as *t*_1/2,*α*_ = *ln*(2)/*α* and *t*_1/2,*β*_ = *ln*(2)/*β*, respectively. These hybrid half-lives were calculated for each simulation replicate using the replicate-specific CL, V_C_, Q, and V_P_ values.

## 3. Results

### 3.1. Preclinical PK of NP-011

Across the evaluated dose ranges (0.0214–0.342 mg/kg in mice, 0.00798–0.171 mg/kg in rats, and 0.00534–0.0854 mg/kg in monkeys), the dose-normalized *C*_max_ and AUC_inf_ values were generally comparable within each species, indicating that the systemic exposure increased approximately in proportion to the dose. The NCA-derived clearance estimates also showed no consistent dose-dependent trend within each species (Tables S3–S5), providing no clear evidence of nonlinear elimination over the evaluated preclinical dose ranges. The mean body-weight-normalized clearance was 0.263–0.457 L/h/kg in mice, 0.442–0.709 L/h/kg in rats, and 0.0735–0.105 L/h/kg in monkeys, indicating lower clearance in monkeys than in rodents. In the monkeys that received 0.0213 mg/kg once daily for 14 d, the dose-normalized AUC_inf_ values on day 14 were comparable to those on day 1. The percentage of AUC extrapolated to infinity was <1% across all preclinical dose groups. Detailed study designs and NCA results are provided in Tables S1 and S3–S5, respectively, and dose-normalized exposure comparisons are shown in Figure S1.

### 3.2. Population PK Model Performance and Evaluation

The plasma concentration–time profiles of NP-011 in all three species were adequately described using a two-compartment model with first-order elimination. The final population PK parameter estimates and corresponding nonparametric bootstrap results are summarized in Table 1.

**Table 1:** Final population pharmacokinetic parameter estimates of NP-011 in mice, rats, and monkeys.

Parameter (Unit)	Mice	Rats	Monkeys
Structural parameters- estimate (%RSE); bootstrap median [95% CI]			
CL (L/h/kg)	0.363 (8.9) 0.351 [0.301–0.416]	0.552 (20.1) 0.600 [0.410–0.774]	0.0904 (3.8) 0.0895 [0.0835–0.0952]
*V_C_* (L/kg)	0.071 (8.2) 0.0685 [0.0592–0.080]	0.0814 (22.2) 0.0896 [0.0589–0.127]	0.0475 (6.0) 0.0466 [0.0415–0.0527]
Q (L/h/kg)	0.0063 (18.9) 0.00614 [0.00449–0.00916]	0.0176 (44.0) 0.0210 [0.0112–0.0463]	0.00342 (9.3) 0.00339 [0.00279–0.00387]
*V_P_* (L/kg)	0.0174 (44.8) 0.0161 [0.00872–0.0301]	0.0627 (22.6) 0.0689 [0.0221–0.122]	0.103 (22.5) 0.104 [0.068–0.145]
Inter-individual variability- ω^2^ estimate (%RSE); median [95% CI]			
IIV on CL	0.0133 (56.9) 0.0117 [0.00071–0.0274]	0.0198 (166.2) 0.0308 [0.00069–0.0953]	0.0022 (131.5) 0.00212 [0.000063–0.00721]
IIV on *V_C_*	–	–	0.0371 (41.8) 0.036 [0.00513–0.0661]
IIV on *V_P_*	0.227 (45.8) 0.218 [0.00653–0.493]	–	–
Residual variability			
Proportional error	0.361 (8.0) 0.354 [0.285–0.404]	0.985 (30.8) 0.762 [0.354–1.325]	0.457 (10.0) 0.433 [0.352–0.516]
Additive error	Fixed to 0	Fixed to 0	Fixed to 0

Notes: Values are presented as the final model estimate (%RSE) and bootstrap median [95% CI] unless otherwise indicated. Structural parameters were normalized to the body weight (kg). CL, clearance; *V_C_*, central volume of distribution; *Q*, intercompartmental clearance; *V_P_*, peripheral volume of distribution; IIV, inter-individual variability; %RSE, relative standard error; ω^2^, variance of the inter-individual random effect; CI, confidence interval from a nonparametric bootstrap (*n* = 1000); – not estimated in the final model. The preclinical studies included 120 mice, 12 rats, and 12 monkeys. Detailed study designs and dose-group allocations are provided in Table S1.

The relative standard errors (%RSEs) of the structural parameter estimates ranged from 3.8 to 44.8%. Inter-individual variability (IIV) was retained on CL and *V_P_* in mice, CL in rats, and CL and *V_C_* in monkeys; the IIV in CL in rats and monkeys was estimated with high uncertainty (%RSE values of 166.2% and 131.5%, respectively). Residual variability was described using a proportional error model, and the additive residual error was fixed to a negligible value for all three species. ETA shrinkage was 7.9% for CL and 43.9% for *V_P_* in mice, 31.6% for CL in rats, and 50.4% for CL and 7.8% for *V_C_* in monkeys (Table S7 and Figure S8).

Goodness-of-fit (GOF) plots showed general agreement between the observed concentrations and both population and individual predictions, and the conditional, weighted residuals showed no apparent systematic patterns over time or across the predicted concentrations (Figures S2–S4). In the visual predictive checks, the final models reproduced the central tendency and variability of the observed concentration–time data across species, with most observed concentration percentiles falling within the simulated 5th–95th percentile ranges (Figures S5–S7). Parameter-correlation analysis identified strong dependencies in the rat model, including correlations of 0.958 between CL and *V_C_* and 0.968 between *Q* and IIV on CL (Figure S9).

In the bootstrap analysis, the median estimates were close to the final model estimates and all estimable parameters fell within the corresponding bootstrap 95% confidence intervals (Table 1). Bootstrap success rates were 98.1%, 99.7%, and 99.6% for mice, rats, and monkeys, respectively, whereas near-boundary estimates occurred in 37.7%, 32.4%, and 46.7% of bootstrap replicates, respectively (Table S7). The 95% confidence intervals for IIV on CL were 0.00069–0.0953 in rats and 0.000063–0.00714 in monkeys.

### 3.3. Prediction of Human PK Parameters by Simple Allometric Scaling

Simple allometric scaling predicted human CL, *V_C_*, *Q*, and *V_P_* values of 3.80 L/h, 2.88 L, 0.25 L/h, and 24.61 L, respectively, for a 70-kg adult (Table 2).

**Figure 1:**
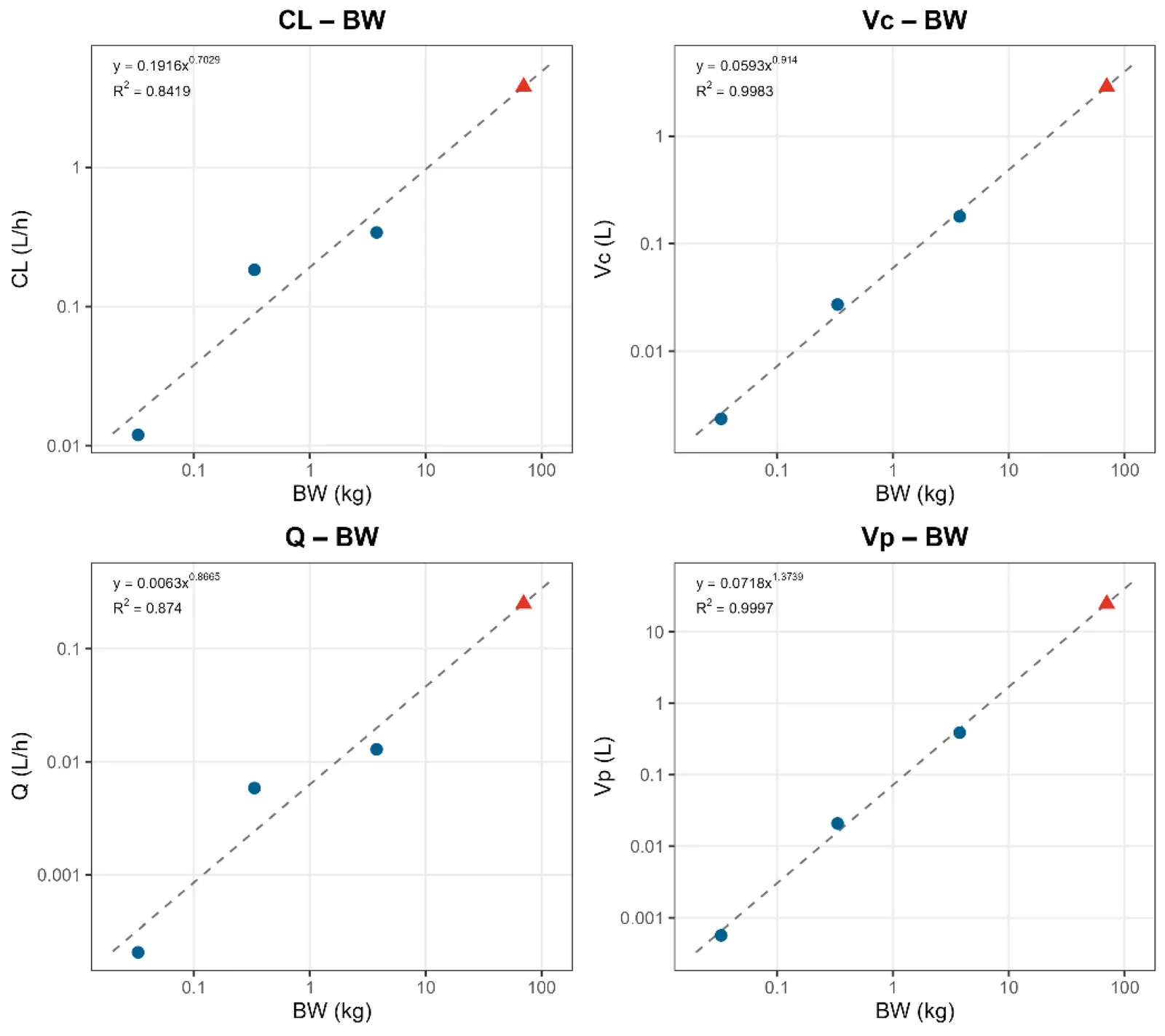
Interspecies allometric scaling of NP-011 PK parameters. Blue circles represent the animal PK parameter estimates, red triangles represent the allometrically predicted values for a 70-kg human, and gray dashed lines represent the fitted allometric relationships. BW, body weight; CL, clearance; VC, central volume of distribution; Q, intercompartmental clearance; VP, peripheral volume of distribution.

The estimated allometric exponents for CL and *Q* were approximately 0.703 and 0.867, respectively (Figure 1), both of which fell within the range of 0.7–1.0 specified by the rule of exponents. Detailed regression characteristics are summarized in Table S10. The 95% confidence interval for the CL exponent was −2.69 to 4.10, and leave-one-species-out estimates ranged from 0.254 to 1.181, indicating sensitivity to the species included in the regression. An additional maximum lifespan potential (MLP) correction was evaluated; the MLP-based results are provided in the Supplementary Information.

### 3.4. Retrospective Evaluation Using Phase I Human PK Data

The human PK profiles of NP-011 were simulated at Phase I, single-dose levels of 0.25, 0.5, 1, 2, and 4 mg using structural parameters predicted by simple allometric scaling. The median predicted AUC_inf_ values were 66, 132, 264, 526, and 1053 ng·h/mL, respectively, and the corresponding median predicted C_max_ values were 87, 173, 345, 695, and 1391 ng/mL. The model-derived median distribution half-life (t_1/2,α_) ranged from 0.492 to 0.498 h across dose levels, whereas the corresponding median terminal half-life (t_1/2,β_) was approximately 72.7 h. The corresponding 90% prediction intervals are listed in Table 3. In the SAD part, exposure was approximately dose-proportional over the 0.25–4 mg range. Power-model slopes were 1.04 (90% CI, 0.98–1.09) for C_max_ and 0.95 (90% CI, 0.89–1.00) for AUC_inf_.

**Figure 2:**
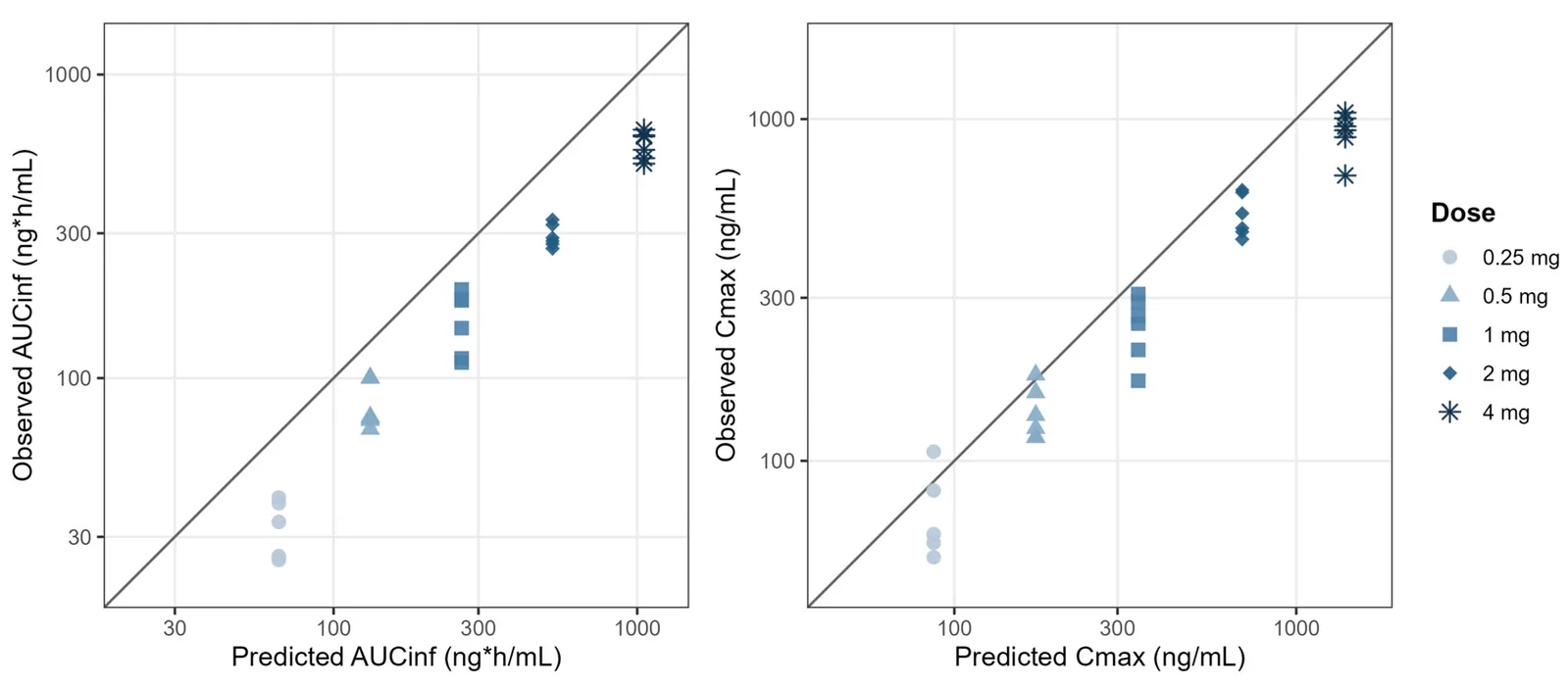
Comparison of observed NP-011 exposure and exposure predicted using simple allometry in the Phase I study. The solid diagonal line represents the line of identity (observed = predicted). Different symbols represent the respective dose groups, as indicated in the figure legend. AUCinf, area under the plasma concentration–time curve from time zero to infinity; Cmax, maximum plasma concentration.

**Figure 3:**
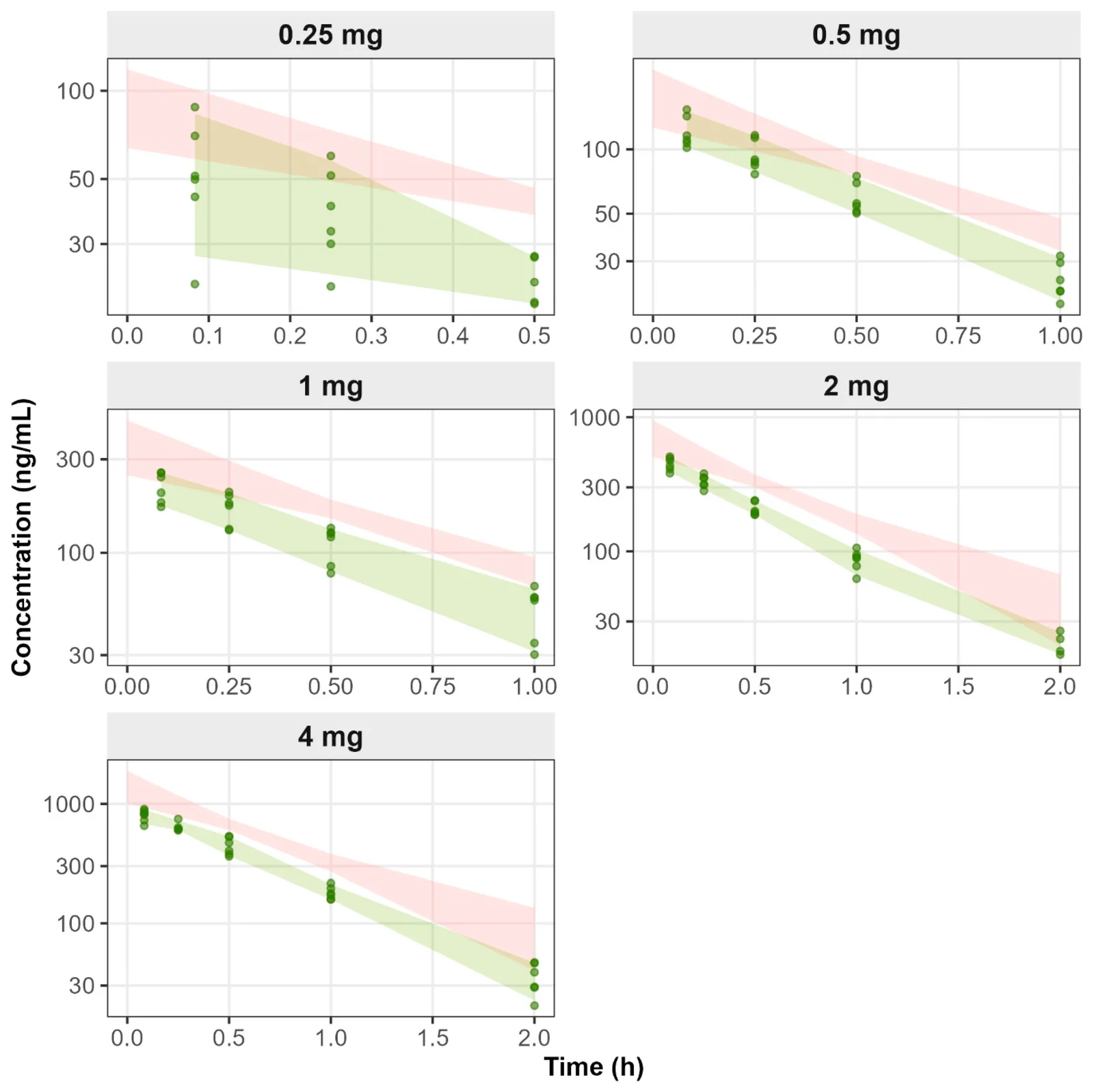
Comparison of NP-011 pharmacokinetic profiles between the Phase I clinical trial and simulations based on simple allometry. The pink shaded area represents the simulated 5th–95th percentile prediction interval; the green shaded area represents the observed 5th–95th percentile interval; and the green points represent the individual observed plasma concentrations.

Across all dose levels, the SA-predicted AUC_inf_ and C_max_ values remained within approximately 2-fold of the observed Phase I values, with C_max_ showing closer agreement than AUC_inf_ (Figure 2). At the dose-group level, the SA-predicted median AUC_inf_ values were 1.62- to 1.96-fold higher than the corresponding observed medians, whereas the predicted median C_max_ values were 1.33- to 1.48-fold higher; the corresponding observed values and quantitative prediction-error metrics are summarized in Table 4. The geometric mean percentage of AUC extrapolated to infinity was 30.6%, 16.9%, 19.6%, 6.29%, and 3.45% at 0.25, 0.5, 1, 2, and 4 mg, respectively. Accordingly, AUC_inf_ estimates at the lower dose levels, particularly at 0.25 and 1 mg, were subject to greater terminal extrapolation and were interpreted with caution. The quantitative assessment of the concentration–time profiles shown in Figure 3 indicated that 21 of 123 observed concentrations (17.1%) were contained within the simulated 5th–95th percentile prediction intervals overall, with dose-specific coverage ranging from 6.7% to 25.0% (Table S11). No neutralizing anti-NP-011 antibodies were detected during the Phase I study. In the MAD part, two participants had pre-existing ADA positivity at baseline and two participants showed transient post-dose ADA positivity at a single time point, with no clear ADA-related effect on the observed concentration data. Repeated once-daily intravenous administration for 7 days showed no evidence of drug accumulation. The accumulation ratios for AUC were 0.91, 0.77, and 0.94, and those for C_max_ were 0.86, 0.83, and 0.99 at 1, 2, and 4 mg, respectively. NP-011 was well tolerated, with all treatment-emergent adverse events being mild in severity.

The MLP-corrected predictions, retained as supplementary sensitivity analyses, showed greater overprediction of AUC_inf_ than the SA predictions. At the dose-group level, the MLP-predicted median AUC_inf_ values were 2.38- to 2.89-fold higher than the corresponding observed medians, with percentage prediction errors ranging from +138% to +189%. For SA, the corresponding values were 1.62- to 1.96-fold and +62% to +96%, respectively. In contrast, C_max_ predictions were unchanged by the MLP correction. Overall, the AFE and AAFE for AUC_inf_ were both 1.79 for SA and 2.64 for MLP, whereas the corresponding values for C_max_ were 1.39 for both methods. These quantitative comparisons indicate closer agreement of SA with the observed human AUC_inf_. Detailed MLP-based results are provided in Tables S12 and S13 and Supplementary Information.

## 4. Discussion

The reliable prediction of human PK from preclinical data is a critical step in the early development of new therapeutics, particularly for novel proteins, such as NP-011, for which no clinical precedent is available. In the current study, human PK parameters were predicted using interspecies allometric scaling and evaluated using observed phase I PK data. The predicted exposures agreed with the observed values within approximately 2-fold, and SA showed closer agreement with the observed Phase I exposure than the MLP-corrected approach in the present retrospective evaluation.

The two-compartment disposition of NP-011 was supported by the observed preclinical concentration–time profiles and the corresponding model diagnostics, including goodness-of-fit plots and visual predictive checks. Across the evaluated preclinical dose ranges, dose-normalized exposure was generally comparable and NCA-derived clearance showed no consistent dose-dependent trend. Consistent with these findings, the Phase I SAD data showed approximately dose-proportional exposure over the 0.25–4 mg range. Collectively, these observations provided no clear evidence of saturable or nonlinear elimination over the exposure ranges evaluated in the present study. Target-mediated disposition, receptor-mediated uptake, proteolytic degradation, plasma protein binding, and other protein-specific disposition processes cannot be excluded mechanistically. However, no clinically relevant nonlinear disposition was evident over the exposure range evaluated. The two-compartment structure is also consistent with that commonly reported for other protein therapeutics [12,13], but this general precedent provides contextual support rather than validation of the NP-011 model.

The choice of species is another determinant of the accuracy of allometric prediction. Mahmood [14] reported that the inclusion of more than two species improved the extrapolation. Tang and Mayersohn [15] found that three-species combinations consisting of mice or rats together with monkeys and dogs provided accurate predictions. They reported that the omission of rats had only a minor effect when data from more than three species were available. The present extrapolation was based on three species, which is consistent with this recommendation, although dogs were not included in the dataset. This difference in the combinations examined by Tang and Mayersohn [15] may have contributed to the residual prediction error, although rats are widely used in preclinical programs due to their availability and practicality [16].

The choice of the scaling method also influences the prediction accuracy. The clearance and volume of distribution are the most frequently extrapolated parameters across species. Volume parameters are generally predicted using SA, whereas several approaches are available for clearance, including SA, ROE corrections based on brain weight (BrW), maximum lifespan potential (MLP), and the monkey liver blood flow method [10]. Of these approaches, SA and ROE are the most widely used. Similar clearance predictions between the two methods have been reported [17,18], and an in-house analysis of 199 drugs found similar proportions of predictions with errors exceeding 2-fold for half-life using SA (approximately 45%) and ROE (approximately 44%) [19]. Therefore, both approaches were evaluated for NP-011.

The estimated exponents for CL and *Q* were 0.703 and 0.867, respectively, placing both parameters within the 0.7–1.0 range for which the ROE criteria recommend MLP correction (Table S8). However, the MLP-corrected predictions showed a greater deviation from the observed exposures than those obtained using SA (Figure S12). This finding is consistent with that of Mahmood [14], who compared SA and ROE in 15 therapeutic proteins and reported that BrW correction was required when the scaling exponents exceeded 1, whereas MLP correction was generally unnecessary for exponents between 0.7 and 1. In that analysis, no correction factor was required for proteins with exponents up to 0.893, whereas the uncertainty remained for values between 0.9 and 0.99. As both exponents estimated for NP-011 were below 0.893, SA was retained for human predictions. Huh et al. [20] reported that clearance was predicted more accurately for macromolecules than for small molecules and attributed these differences to their distinct elimination pathways. Small molecules are predominantly eliminated through hepatic phase I and II metabolism, whereas proteins are cleared primarily through non-specific proteolytic degradation.

The allometric exponents were derived from only three species, resulting in wide confidence intervals (e.g., 95% CI for the CL exponent, −2.69 to 4.10) and marked sensitivity to the inclusion of individual species (leave-one-species-out CL exponents, 0.25–1.18). This is particularly relevant to CL and *Q*, because the decision to apply the ROE-based MLP correction depends directly on the estimated allometric exponent. The estimated CL exponent (0.703) was close to the lower threshold for applying this correction, making the choice of scaling method sensitive to uncertainty in the exponent. This uncertainty, together with uncertainty in the animal population PK parameter estimates and the assumed human body weight, was not formally propagated into the human predictions. Accordingly, the reported simulation intervals should be interpreted as conditional intervals under the selected scaling and variability assumptions rather than as comprehensive prediction intervals incorporating all sources of uncertainty. Monkey-derived inter-individual variability was transferred to the human simulations as a pragmatic assumption in the absence of empirically estimated human variability. Given the limited precision of the animal IIV estimates, this assumption should not be interpreted as a biologically established estimate of human IIV. The model diagnostics further indicated limited identifiability of some random-effects and structural parameters. In particular, ETA shrinkage was relatively high for CL in monkeys (50.4%) and *V_P_* in mice (43.9%), and strong parameter correlations were observed in the rat model, including CL–*V_C_* and Q–IIV on CL. These findings, together with the frequent near-boundary bootstrap estimates, suggest that the corresponding variability and correlated parameter estimates should be interpreted cautiously. A fixed-effects prediction using the typical structural parameters reproduced the medians of the variability-based simulations to within 0.7% across all dose levels, indicating that the central tendency of the predictions was insensitive to the transferred inter-individual variability. For the rat model, the lack of improvement after freely estimating the additive residual component, together with its boundary behavior, supported retention of the proportional residual-error model despite the comparatively large residual variability.

The simulated 5th–95th percentile intervals showed limited coverage of the observed concentrations. This likely reflects both the systematic overprediction of exposure and the relatively narrow simulated intervals, which incorporated inter-individual variability only for CL and *V_C_*, while *Q* and *V_P_* were fixed, and did not include residual variability. Accordingly, these intervals primarily represent parameter-driven variability rather than the full variability observed in the clinical data. The comparison should therefore be interpreted primarily in terms of central tendency, based on the agreement between median predicted and observed exposure, rather than as evidence of adequate prediction-interval coverage.

The model-derived distribution half-life was approximately 0.5 h, and the limited sampling density during the early post-dose period may have reduced the precision with which the rapid distribution phase could be characterized. Similarly, the model-derived terminal half-life of approximately 72.7 h should be interpreted with caution. The available clinical sampling duration did not adequately characterize such a prolonged terminal phase, and this terminal half-life was inferred from the extrapolated two-compartment structural parameters rather than directly estimated from the terminal phase of the observed Phase I concentration–time data. The limited clinical sampling duration also affected the reliability of the observed AUC_inf_ estimates at the lower dose levels, where a substantial proportion of the total exposure was derived from terminal extrapolation. The observed ADA findings were limited to a small number of transient or pre-existing positive results, and no neutralizing antibodies were detected. Immunogenicity was therefore not considered a major contributor to the observed PK profiles and was not incorporated into the model. This limitation was most pronounced at the lower dose levels, particularly at 0.25 and 1 mg, where a greater proportion of total exposure was derived from terminal extrapolation. Accordingly, the comparisons of predicted and observed AUC_inf_ at these dose levels should be interpreted with greater caution than those at the higher dose levels.

## 5. Conclusions

A population PK model of NP-011 was successfully developed using preclinical data from mice, rats, and monkeys. Human PK parameters were subsequently extrapolated using interspecies allometric scaling and retrospectively evaluated using Phase I PK data. SA predicted human exposure with reasonable accuracy and showed better agreement with the observed clinical data than MLP correction. These findings support the use of SA as a practical approach for PK of NP-011 in healthy adults in the present dataset, although broader generalization, including to patients with MASH, requires further evaluation.

## Figures and Tables

**Table 2:** Human pharmacokinetic parameters of NP-011 extrapolated from animal data using simple allometry.

Parameter	Predicted Value in a 70-kg Adult
CL (L/h)	3.80
*V_C_* (L)	2.88
*Q* (L/h)	0.25
*V_P_* (L)	24.61

CL, clearance; V*_C_*, central volume of distribution; *Q*, intercompartment clearance; V*_P_*, peripheral volume of distribution.

**Table 3:** Human pharmacokinetic parameters of NP-011 predicted using simple allometry.

Dose (mg)	Median AUC_inf_ (ng/mL*h)	90% Prediction Interval AUC_inf_ (ng/mL*h)	Median C_max_ (ng/mL)	90% Prediction Interval for C_max_ (ng/mL)	Model-Derived t_1/2,α_ (h), Median (90% PI)	Model-Derived t_1/2,β_ (h), Median (90% PI)
0.25	66	61–71	87	64–118	0.494 (0.358–0.679)	72.73 (72.40–73.09)
0.5	132	122–142	173	126–236	0.495 (0.357–0.686)	72.74 (72.40–73.10)
1	264	244–285	345	249–475	0.498 (0.357–0.691)	72.74 (72.40–73.10)
2	526	489–569	695	507–949	0.492 (0.358–0.683)	72.73 (72.40–73.10)
4	1053	976–1139	1391	1011–1886	0.492 (0.360–0.681)	72.73 (72.40–73.10)

AUC, area under the plasma concentration–time curve; C_max_, maximum plasma concentration; t_1/2,α_, model-derived distribution half-life; t_1/2,β_, model-derived terminal half-life; PI, prediction interval. The hybrid half-lives were calculated for each simulation replicate from the corresponding CL, *V_C_*, *Q*, and *V_P_* values.

**Table 4:** Comparison of observed and SA predicted Phase I exposure metrics by dose level.

Dose (mg)	Observed AUC_inf_, Median (n)	P/O Ratio for AUC_inf_	%PE for AUC_inf_	Observed C_max_ Median (ng/mL)	P/O Ratio for C_max_	%PE for C_max_
0.25	34 (5)	1.96	96	61	1.42	42
0.5	74 (6)	1.78	78	130	1.33	33
1	163 (6)	1.62	62	259	1.33	33
2	286 (6)	1.84	84	505	1.38	38
4	595 (6)	1.77	77	940	1.48	48

Predicted median values and their corresponding 90% prediction intervals are reported in Table 3. P/O, predicted-to-observed ratio; %PE, percentage prediction error. Comparisons were performed using dose-group summary values, with observed cohort medians compared with the corresponding median predictions.

## Data Availability

The data supporting the findings of this study are available within the article and its Supplementary Materials. The individual-level preclinical and clinical data are not publicly available because of proprietary and participant-confidentiality restrictions but may be available from the corresponding author upon reasonable request and subject to applicable ethical, legal, and data-sharing requirements.

## Supplementary Materials

The following supporting information can be downloaded at: https://www.mdpi.com/article/10.3390/pharmaceutics18091194/s1, Supplementary Methods; Supplementary Results; Table S1: Design and sampling characteristics of the preclinical pharmacokinetic studies; Table S2: Phase I study design, participant allocation, and PK sampling scheme; Table S3: PK parameters of NP-011 in mice through NCA; Table S4: PK parameters of NP-011 in rats through NCA; Table S5: PK parameters of NP-011 in monkeys through NCA; Table S6: Specification and estimation details of the final population pharmacokinetic models; Table S7: Bootstrap performance and shrinkage diagnostics of the final population PK models; Table S8: Modification of simple allometry; Table S9: Brain weight of species; Table S10: Allometric regression and sensitivity analyses for human pharmacokinetic extrapolation; Table S11: Coverage of observed Phase I concentrations within the simulated 5th–95th percentile prediction intervals under the SA and MLP methods; Table S12: Human-predicted PK parameters of NP-011 using maximum lifespan potential; Table S13: Comparison of observed and MLP-corrected predicted Phase I exposure metrics by dose level; Figure S1: Dose-normalized PK parameters across species; Figure S2: Goodness-of-fit diagnostics for the final population pharmacokinetic model of NP-011 in mice; Figure S3: Goodness-of-fit diagnostics for the final population pharmacokinetic model of NP-011 in rats; Figure S4: Goodness-of-fit diagnostics for the final population pharmacokinetic model of NP-011 in monkeys; Figure S5: Visual predictive check of the final population pharmacokinetic model in mice, stratified by dose; Figure S6: Visual predictive check of the final population pharmacokinetic model in rats, stratified by dose; Figure S7: Visual predictive check of the final population pharmacokinetic model in monkeys, stratified by dose; Figure S8: ETA distributions and shrinkage for the final population pharmacokinetic models; Figure S9: Parameter-correlation matrices for the final population pharmacokinetic models in mice, rats, and monkeys; Figure S10: Residual-error sensitivity analysis for the rat population pharmacokinetic model; Figure S11: Interspecies allometric scaling of NP-011 pharmacokinetic parameters using maximum lifespan potential correction; Figure S12: Comparison of observed and MLP-predicted NP-011 exposure in the Phase I study; Figure S13: Comparison of NP-011 pharmacokinetic profiles between the Phase I clinical trial and simulations based on maximum lifespan potential. The NONMEM simulation control stream (SIM_SA.mod), the R script used to reproduce Figure 3 (Figure3_SA_profile_with_obs.R), and the aggregate dataset containing the observed and simulated summary profiles (aggregate_Figure3_SA_profile.csv) are provided as separate supplementary files.

## Abbreviations

The following abbreviations are used in this manuscript:

MASH	Metabolic dysfunction-associated steatohepatitis
MASLD	Metabolic dysfunction-associated steatotic liver disease
PK	Pharmacokinetics
AUC	Area under the plasma concentration–time curve
BrW	Brain weight
CL	Clearance
GOF	Goodness-of-fit
IIV	Inter-individual variability
IV	Intravenous
MLP	Maximum lifespan potential
SA	Simple allometry
ROE	Rule of exponents
NCA	Noncompartmental analysis
OFV	Objective function value
VPC	Visual predictive check
