# Supplementary material for: Population Pharmacokinetics and Extrapolation of NP-011 from Animal to Humans: A Novel Therapeutic Candidate Protein for Metabolic Dysfunction-Associated Steatohepatitis
Source: Pharmaceutics. 2026 Sep 21;18(9):1194. doi: 10.3390/pharmaceutics18091194 (PMC13610530; doi:10.3390/pharmaceutics18091194)
Supplement: Supplementary file 1 [file pharmaceutics-18-01194-s001.zip › pharmaceutics-4528146-supplementary.pdf]

Supplementary Methods

Data structure and variable definitions

The NONMEM modeling datasets contained standard variables for individual identification, dosing, and observation records, including ID, TIME, AMT, DV, CMT, EVID, and MDV. TIME was expressed in hours. In the preclinical datasets, dose amounts (AMT) were expressed on a body-weight-normalized basis in µg/kg, and plasma concentrations were measured in ng/mL. The dependent variable used for model estimation was the natural-log-transformed plasma concentration. Accordingly, the structural parameters were estimated on a body-weight-normalized basis, with CL and Q expressed in L/h/kg and V_C and V_P expressed in L/kg.

Bioanalytical assay validation and BLQ handling

NP-011 concentrations in preclinical plasma samples were quantified using validated ELISA methods. The preclinical assays were evaluated for calibration and quantification ranges, intra- and inter-assay accuracy and precision, dilutional linearity, hook effect, and stability according to predefined acceptance criteria. Clinical plasma concentrations were quantified using a separately validated ELISA with an LLOQ of 15.62 ng/mL. Detailed quantitative assessments of selectivity, recovery or matrix effects, and cross-reactivity with endogenous human MFG-E8 are contained in the corresponding bioanalytical validation documentation and are not reproduced in full here.

For NCA, BLQ concentrations at predose and before T_{max} were set to zero, whereas BLQ observations after T_{max} were treated as missing or excluded. For population PK modeling, BLQ observations were excluded from the modeling dataset using an M1 approach; no likelihood-based M3 or M4 method was applied.

Population pharmacokinetic model specification and evaluation

Plasma concentration–time data for NP-011 were described separately in mice, rats, and monkeys using two-compartment disposition models implemented in NONMEM with ADVAN6/TRANS1 and user-defined differential equations. The model was parameterized in terms of clearance (CL), central volume of distribution (V_C), intercompartmental clearance Q, and peripheral volume of distribution (V_P). The amounts of NP-011 in the central (A_C) and peripheral (A_P) compartments were described by:

$$\frac{dA_c}{dt} = -\left(\frac{\mathrm{CL}}{V_C} + \frac{\mathrm{Q}}{V_C}\right) A_c + \frac{\mathrm{Q}}{V_P} A_P$$

$$\frac{dA_P}{dt} = \frac{\mathrm{Q}}{V_C} A_c + \frac{\mathrm{Q}}{V_P} A_P$$

The model-predicted plasma concentration was calculated as

$$C = \frac{A_c}{V_C}$$

Equivalently, the micro-rate constants were defined as

$$k_{10} = \frac{CL}{V_C}, \quad k_{12} = \frac{Q}{V_C}, \quad k_{21} = \frac{Q}{V_P}$$

For intravenous bolus administration, the dose was entered directly into the central compartment. Inter-individual variability (IIV) was described using an exponential random-effects model:

$$P_i = P_{TV} \exp(\eta_i)$$

where P_i is the individual parameter value, P_{TV} is the typical population value, and η_i is a normally distributed random effect with a mean of zero and variance ω^2. IIV was retained on CL and V_P in mice, CL in rats, and CL and V_C in monkeys. The OMEGA matrices were

specified as diagonal, and no covariance between random effects was estimated.

Residual unexplained variability was modeled on the natural-log-transformed concentration scale. A proportional residual-error component was retained in the final models, whereas the additive component was fixed to a negligible value when it could not be estimated reliably. Parameters were estimated using the first-order conditional estimation method with interaction (FOCE-I). One- and two-compartment structural models were compared using the objective function value (OFV). For nested-model comparisons, statistical significance was assessed using the likelihood-ratio test, with the degrees of freedom corresponding to the difference in the number of estimated parameters between models and the corresponding chi-square critical value at $\alpha = 0.05$.

Initial parameter estimates were obtained from preceding model-development runs. The initial and final parameter estimates, fixed parameters, and the minimization and covariance-step status for each species are summarized in Table S6. Both the minimization and covariance steps were completed successfully for the final models.

Model adequacy and parameter precision were evaluated using goodness-of-fit diagnostics, nonparametric bootstrap analyses, ETA distributions and shrinkage, parameter-correlation matrices, and dose-stratified visual predictive checks. The bootstrap analysis was based on 1,000 replicates per species and resampled individual animals rather than individual records, thereby preserving the complete within-animal observation sequence in each bootstrap replicate. Bootstrap success rates, near-boundary estimates, failure modes, and nonparametric 95% confidence intervals are summarized in Table S7.

For the rat model, the adequacy of the residual-error structure was further examined in a sensitivity analysis in which the additive residual component was freely estimated and compared with the final model.

Maximum life-span potential (MLP)-corrected allometric scaling

As a supplementary sensitivity analysis, human clearance parameters were additionally estimated using maximum life-span potential (MLP)-corrected allometric scaling. Clearance (CL) and inter-compartmental clearance (*Q*) were corrected using the MLP method, whereas the central volume of distribution (V_c) and peripheral volume of distribution (V_p) were extrapolated using simple allometry.

The MLP of each species was calculated according to the following equation:

$$MLP(year) = 185.4 * (BrW^{0.636} * BW^{-0.225})$$

where BrW and BW represent brain weight and body weight (g), respectively. Species-specific brain weights are summarized in Table S9.

Human clearance parameters were then estimated using the following MLP-corrected allometric equation:

$$CL_{human} = CL_{animal} * \left(\frac{\mathrm{BW}_{human}}{\mathrm{BW}_{animal}}\right)^{b} * \left(\frac{\mathrm{MLP}_{animal}}{\mathrm{MLP}_{human}}\right)$$

where b is the allometric exponent estimated from the simple allometric regression. The resulting human PK parameters were incorporated into the final population pharmacokinetic model, and human concentration-time profiles were simulated using the same procedure described for the primary simple-allometry analysis.

Supplementary Results

Population pharmacokinetic model diagnostics and sensitivity analyses

Comparison of the structural models showed that the two-compartment model reduced the OFV by 154.7, 95.8, and 407.6 relative to the one-compartment model in mice, rats, and monkeys, respectively. In each species, the net increase in the number of estimated parameters was two, corresponding to 2 degrees of freedom, and the OFV reductions substantially exceeded the chi-square critical value of 5.99 at $\alpha = 0.05$.

The final population PK models showed high bootstrap completion rates of 98.1%, 99.7%, and 99.6% for mice, rats, and monkeys, respectively. Near-boundary estimates occurred in 37.7%, 32.4%, and 46.7% of bootstrap replicates, respectively. The bootstrap confidence intervals for several IIV parameters extended close to zero, particularly for CL in rats and monkeys, indicating limited precision of these variability estimates.

ETA shrinkage was 7.9% for CL and 43.9% for V_P in mice, 31.6% for CL in rats, and 50.4% for CL and 7.8% for V_C in monkeys. Parameter-correlation analysis identified strong dependencies in the rat model, including correlations of 0.958 between CL and V_C and 0.968 between Q and IIV on CL. Corresponding ETA distributions and parameter-correlation matrices are presented in Figures S8 and S9, respectively, and shrinkage estimates are summarized in Table S7.

In the rat residual-error sensitivity analysis, freely estimating the additive residual component did not improve model fit compared with the final proportional-error model (ΔOFV = +1.621; OFV, 70.503 vs. 68.882). The additive component approached its lower boundary, and low-concentration predictions were not materially improved (Figure S10). These findings supported

retention of the final residual-error specification.

Human PK prediction using MLP-corrected allometric scaling

The estimated allometric exponents for CL and Q were 0.703 and 0.867, respectively, meeting the ROE criterion for application of the MLP correction. MLP-corrected allometric scaling predicted human CL and Q values of 2.58 L/h and 0.17 L/h, respectively, whereas V_c and V_p remained unchanged at 2.88 L and 24.61 L. The corresponding interspecies scaling relationships are presented in Figure S11.

Human PK profiles simulated using the MLP-derived parameters are shown in Figure S12. The predicted median AUC_{inf} values were 97, 194, 389, 775, and 1552 ng·h/mL at the 0.25, 0.5, 1, 2, and 4 mg dose levels, respectively. The corresponding median C_{max} values were 87, 173, 345, 695, and 1391 ng/mL. The model-derived median distribution half-life ($t_{1/2,\alpha}$) ranged from 0.725 to 0.734 h across dose levels, whereas the corresponding median terminal half-life ($t_{1/2,\beta}$) was approximately 107.3 h (Table S10).

Comparison of simulated concentration-time profiles with the observed Phase I data is presented in Figure S13. Relative to the simple allometry predictions, the MLP-corrected approach yielded higher predicted AUC_{inf} values, whereas the predicted C_{max} values were unchanged.

Reproducibility of the human PK simulation

The simple-allometry human PK simulations were performed using 1,000 simulation replicates with fixed random seeds of 12345 and 54321. The corresponding NONMEM simulation control stream (SIM_SA.mod), the R script used to reproduce Figure 3 (Figure3_SA_profile_with_obs.R), and an aggregate dataset containing the observed and simulated summary profiles used for Figure 3 (aggregate_Figure3_SA_profile.csv) are provided as separate supplementary files.

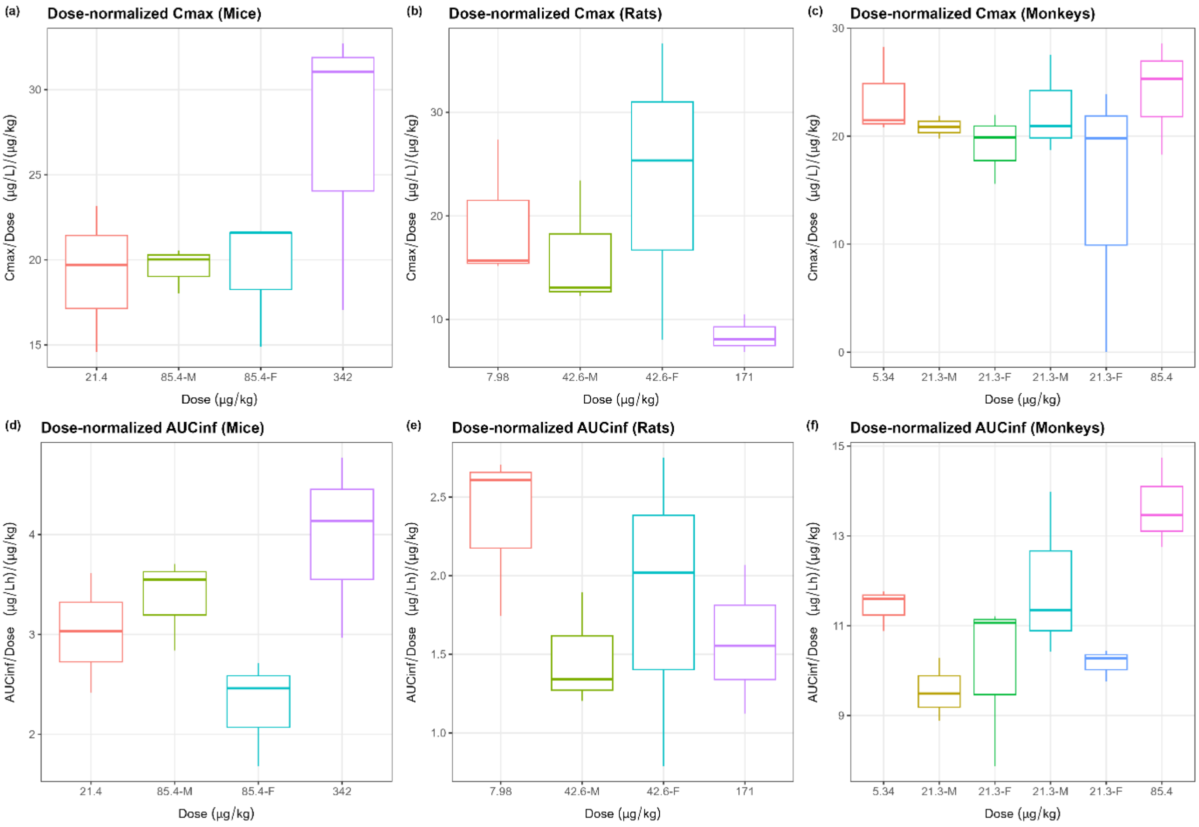

Figure S1. Dose-normalized PK parameters across species.; M-male, F-female.

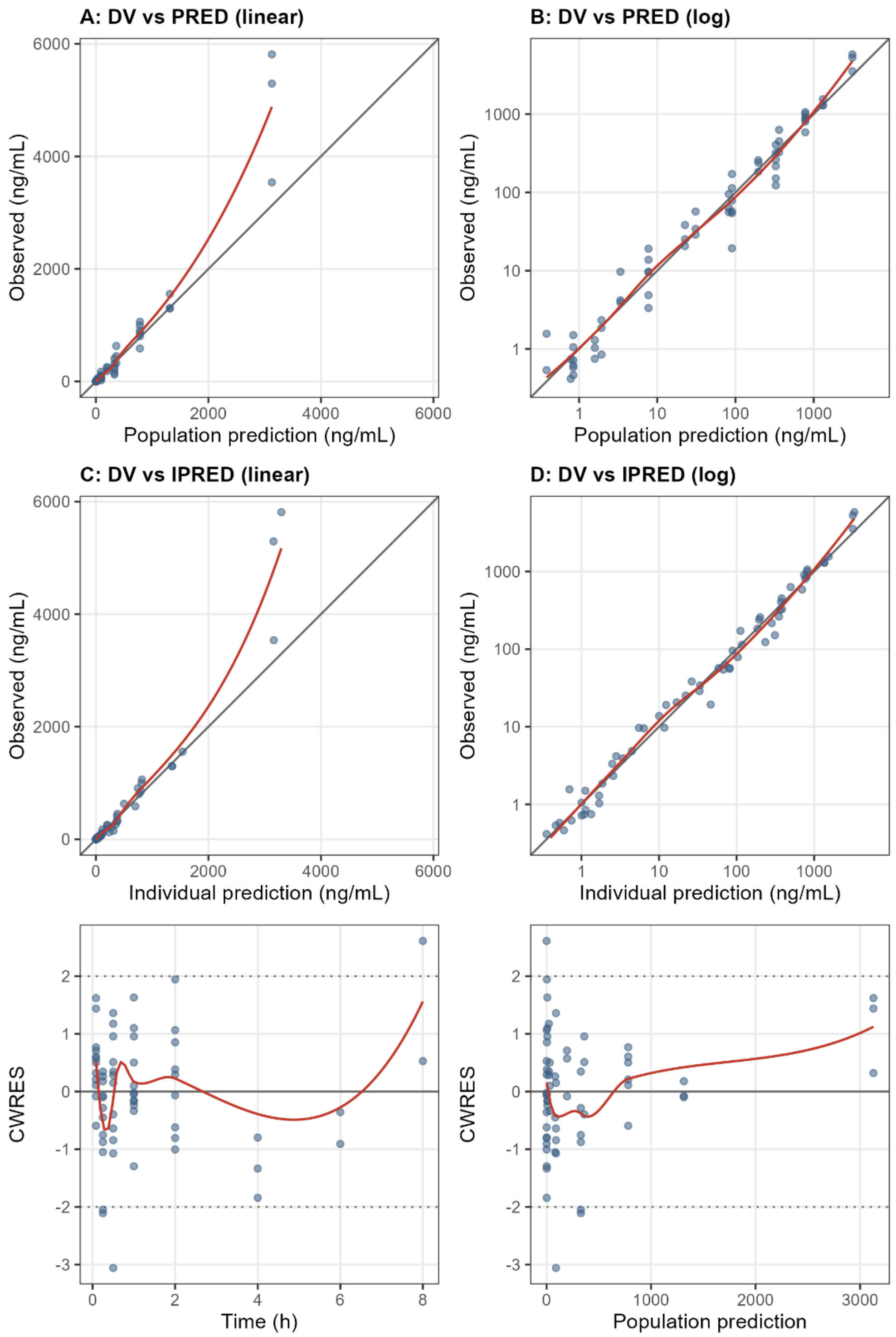

Figure S2. Goodness-of-fit diagnostics for the final population pharmacokinetic model of NP-011 in mice. Panels show observed versus population predictions and individual predictions on linear and logarithmic scales, and conditional weighted residuals versus time and population predictions. The solid black line represents the line of identity, the red line

represents a locally smoothed trend, and the dotted horizontal lines indicate CWRES values of ± 2. IPRED, individual prediction; PRED, population prediction; CWRES, conditional weighted residual.

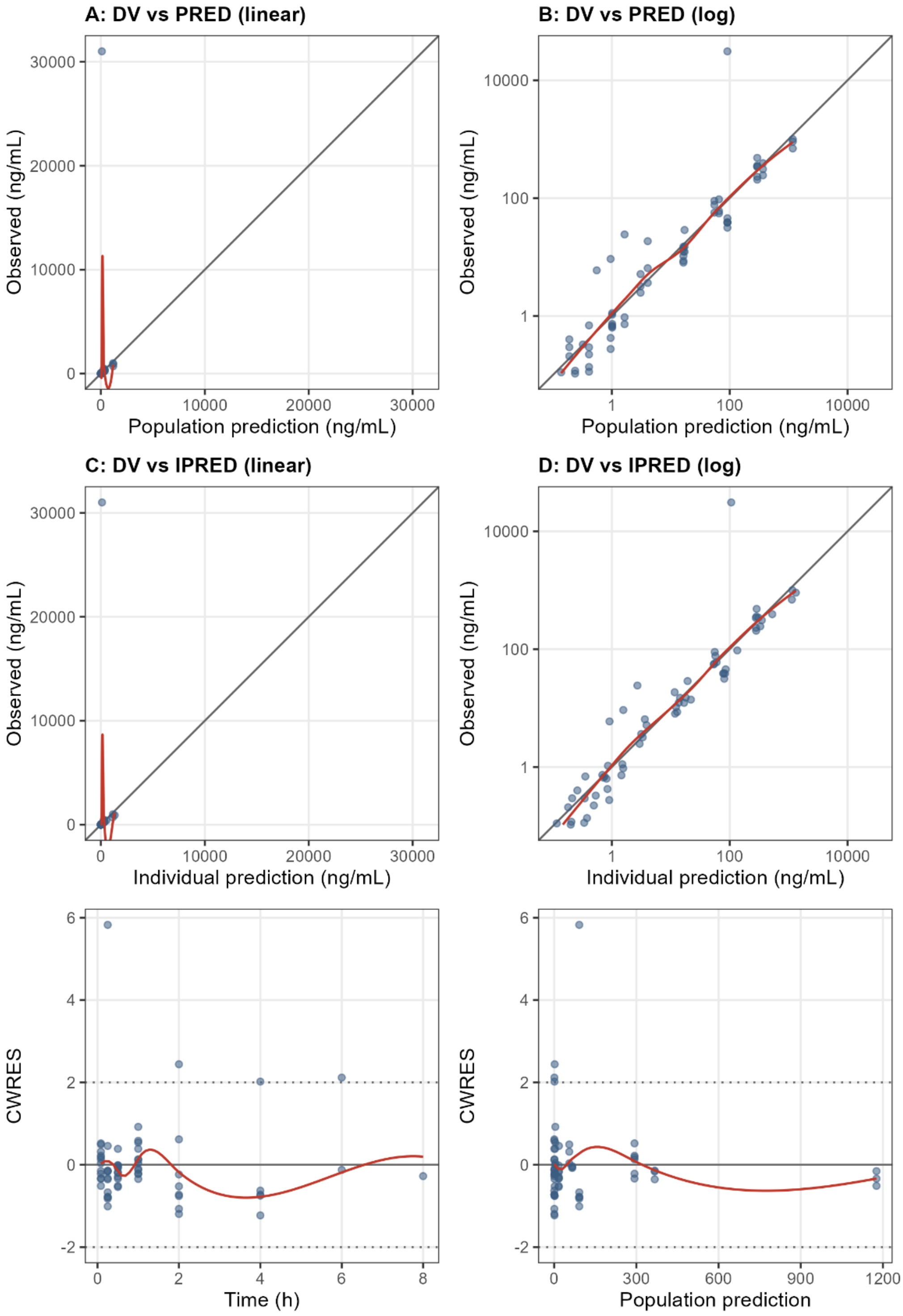

Figure S3. Goodness-of-fit diagnostics for the final population pharmacokinetic model of NP-011 in rats. Panels show observed versus population predictions and individual predictions on linear and logarithmic scales, and conditional weighted residuals versus time and population predictions. The solid black line represents the line of identity, the red line

represents a locally smoothed trend, and the dotted horizontal lines indicate CWRES values of ± 2. IPRED, individual prediction; PRED, population prediction; CWRES, conditional weighted residual.

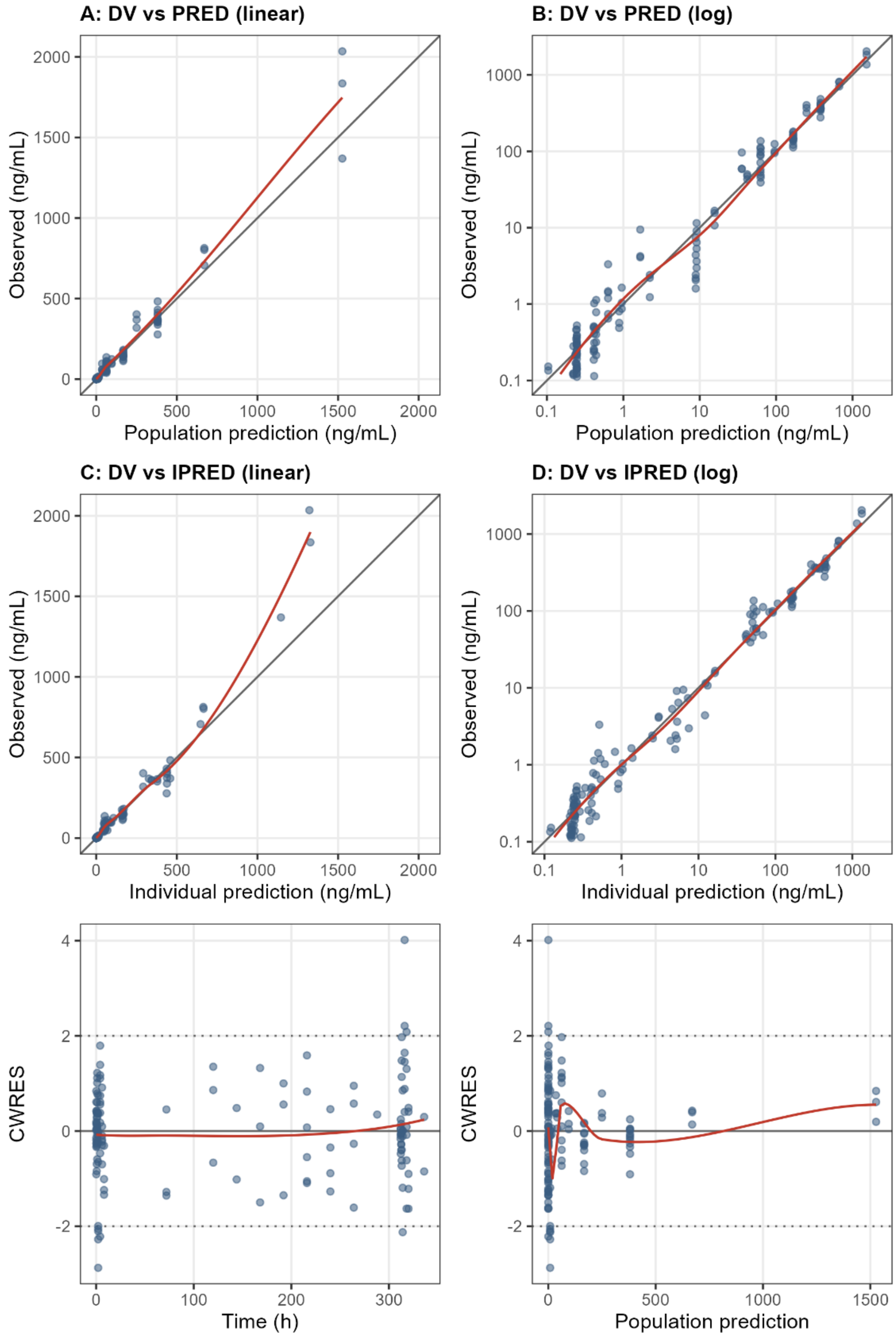

Figure S4. Goodness-of-fit diagnostics for the final population pharmacokinetic model of NP-011 in monkeys. Panels show observed versus population predictions and individual predictions on linear and logarithmic scales, and conditional weighted residuals versus time

and population predictions. The solid black line represents the line of identity, the red line represents a locally smoothed trend, and the dotted horizontal lines indicate CWRES values of ± 2. IPRED, individual prediction; PRED, population prediction; CWRES, conditional weighted residual.

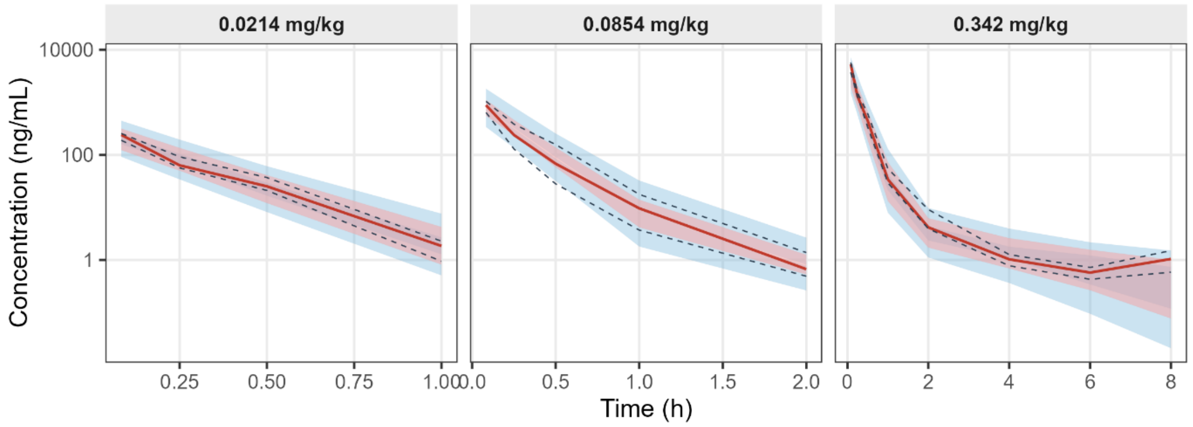

Figure S5. Visual predictive check of the final population pharmacokinetic model in mice, stratified by dose. The solid red line represents the observed median, and the thin dashed lines represent the observed 5th and 95th percentiles. The pink band represents the 95% confidence interval of the simulated median, whereas the blue bands represent the 95% confidence intervals of the simulated 5th and 95th percentiles based on 1,000 simulation replicates. Concentrations were back-transformed from the natural-log scale used for model estimation and are displayed in ng/mL on a logarithmic (base-10) y-axis.

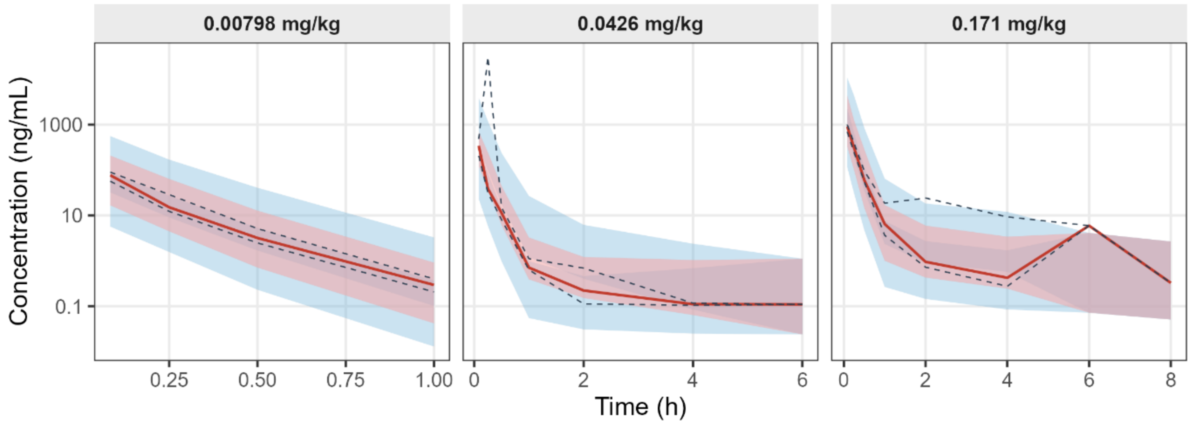

Figure S6. Visual predictive check of the final population pharmacokinetic model in rats, stratified by dose. The solid red line represents the observed median, and the thin dashed lines represent the observed 5th and 95th percentiles. The pink band represents the 95% confidence interval of the simulated median, whereas the blue bands represent the 95% confidence intervals of the simulated 5th and 95th percentiles based on 1,000 simulation replicates. Concentrations were back-transformed from the natural-log scale used for model estimation and are displayed in ng/mL on a logarithmic (base-10) y-axis.

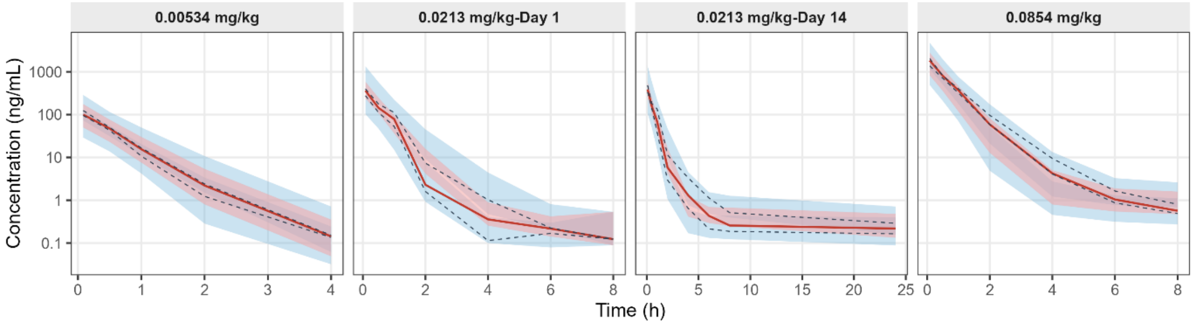

Figure S7. Visual predictive check of the final population pharmacokinetic model in monkeys, stratified by dose. The solid red line represents the observed median, and the thin dashed lines represent the observed 5th and 95th percentiles. The pink band represents the 95% confidence interval of the simulated median, whereas the blue bands represent the 95% confidence intervals of the simulated 5th and 95th percentiles based on 1,000 simulation replicates. Concentrations were back-transformed from the natural-log scale used for model estimation and are displayed in ng/mL on a logarithmic (base-10) y-axis. For the once-daily repeated-dose cohort (0.0213 mg/kg), the intensive post-dose profiles on Days 1 and 14 are shown in separate panels, with Day 14 time re-based relative to the Day 14 dose.

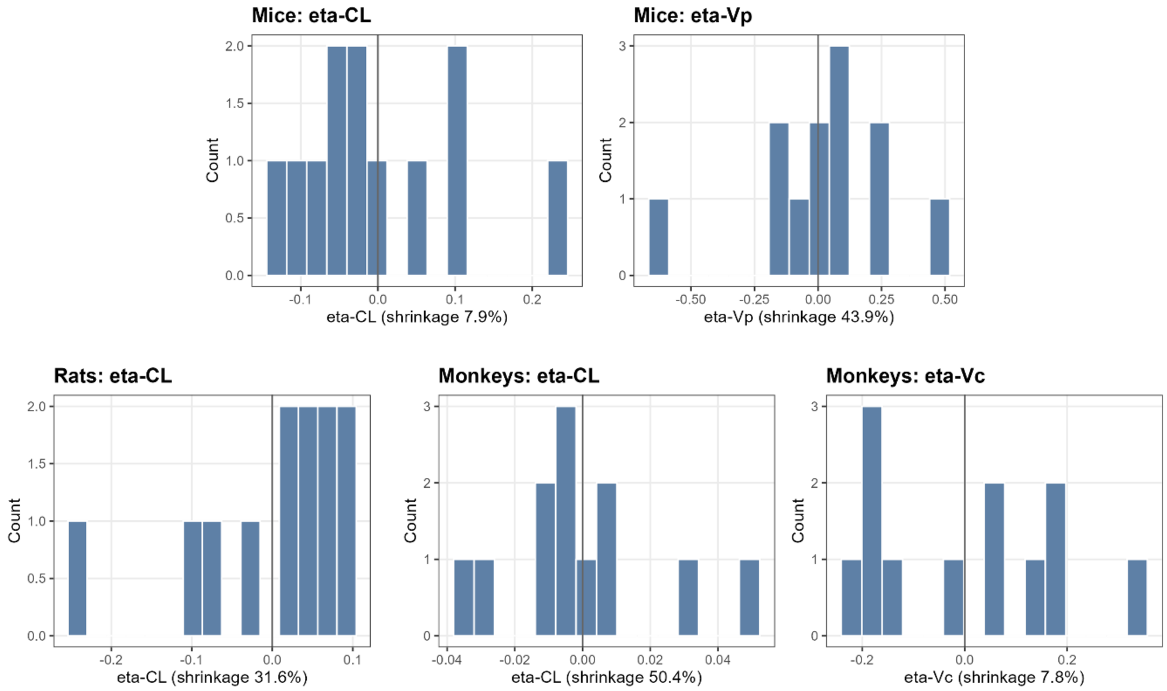

Figure S8. ETA distributions and shrinkage for the final population pharmacokinetic models. Histograms show ETA distributions for CL and V_P in mice, CL in rats, and CL and V_C in monkeys. Vertical lines indicate zero; shrinkage values are shown on the x-axis labels.

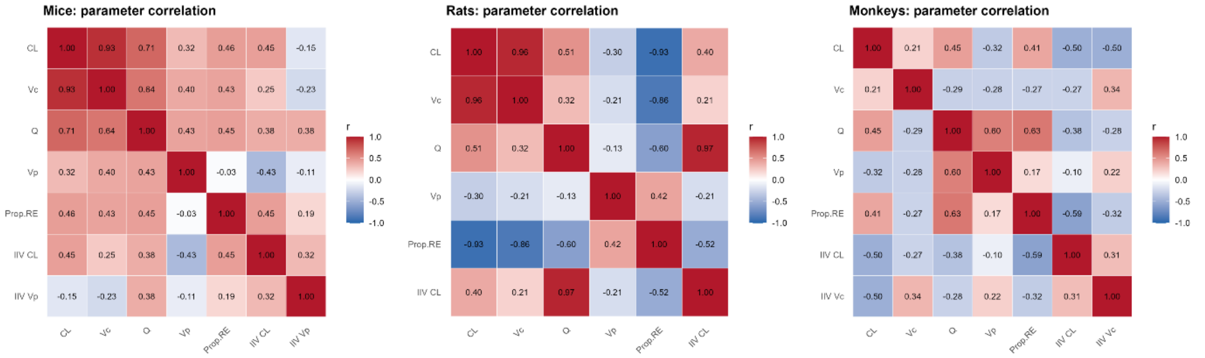

Figure S9. Parameter-correlation matrices for the final population pharmacokinetic models in mice, rats, and monkeys. Values indicate correlation coefficients from the covariance matrix of the final NONMEM estimates. CL, clearance; V_C, central volume of distribution; Q,

intercompartmental clearance; V_P, peripheral volume of distribution; Prop.RE, proportional residual error; IIV, inter-individual variability.

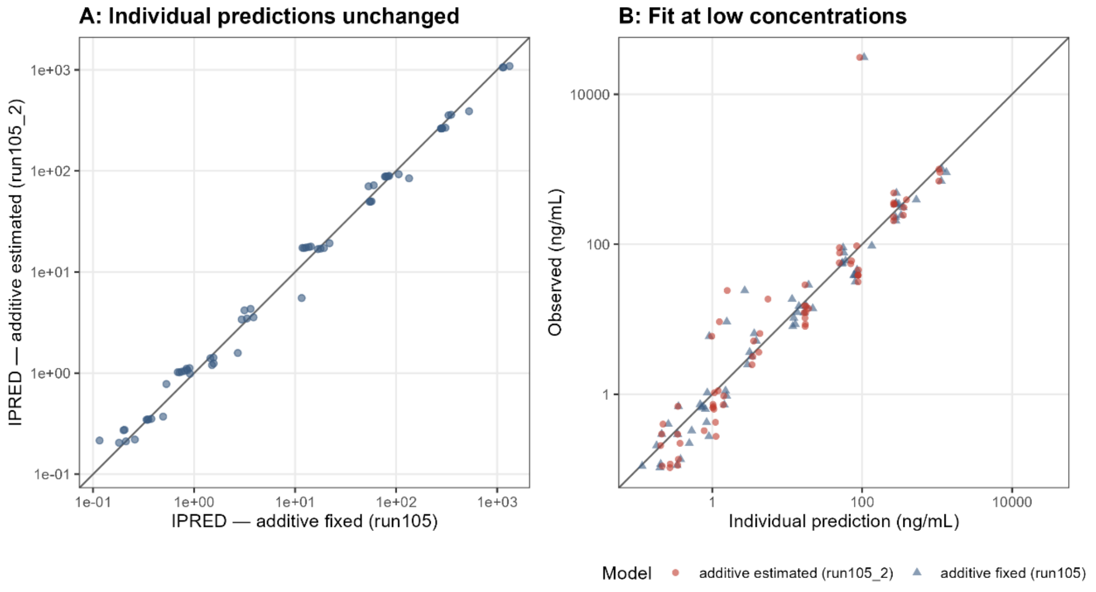

Figure S10. Residual-error sensitivity analysis for the rat population pharmacokinetic model. (**A**) Comparison of individual predictions between the final and alternative residual-error models. (**B**) Observed versus individual-predicted concentrations at low concentrations.

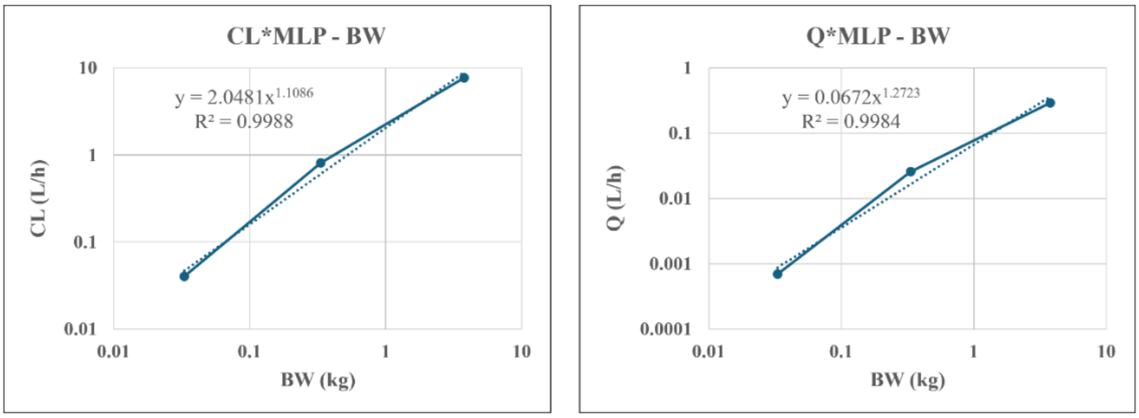

Figure S11. Interspecies allometric scaling of NP-011 pharmacokinetic parameters using maximum life-span potential correction. BW, body weight; CL, clearance; *Q*, inter-compartmental clearance.

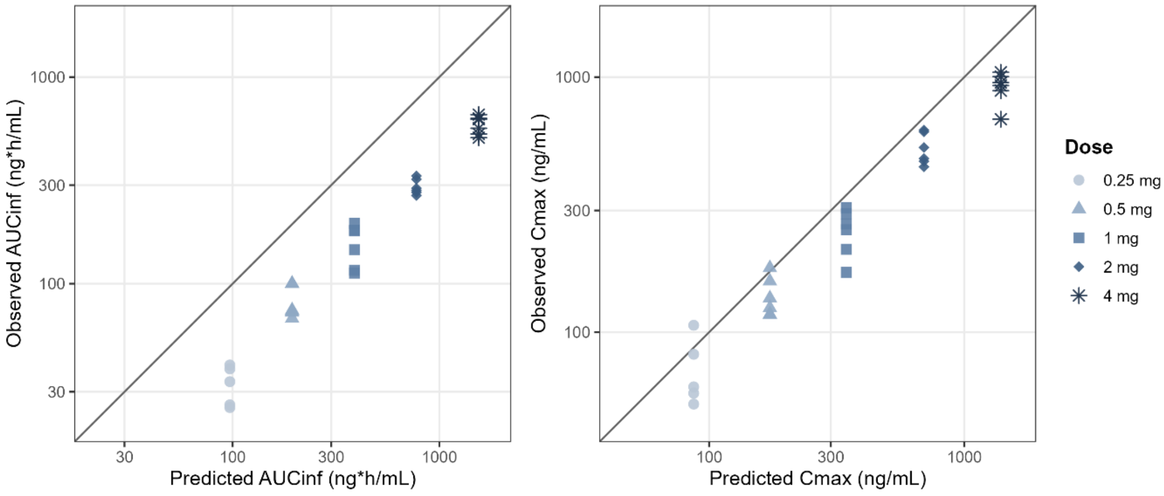

Figure S12. Comparison of observed and MLP predicted NP-011 exposure in the Phase I study. AUC_{inf}, area under the plasma concentration-time curve from time zero to infinity; C_{max}, maximum plasma concentration.

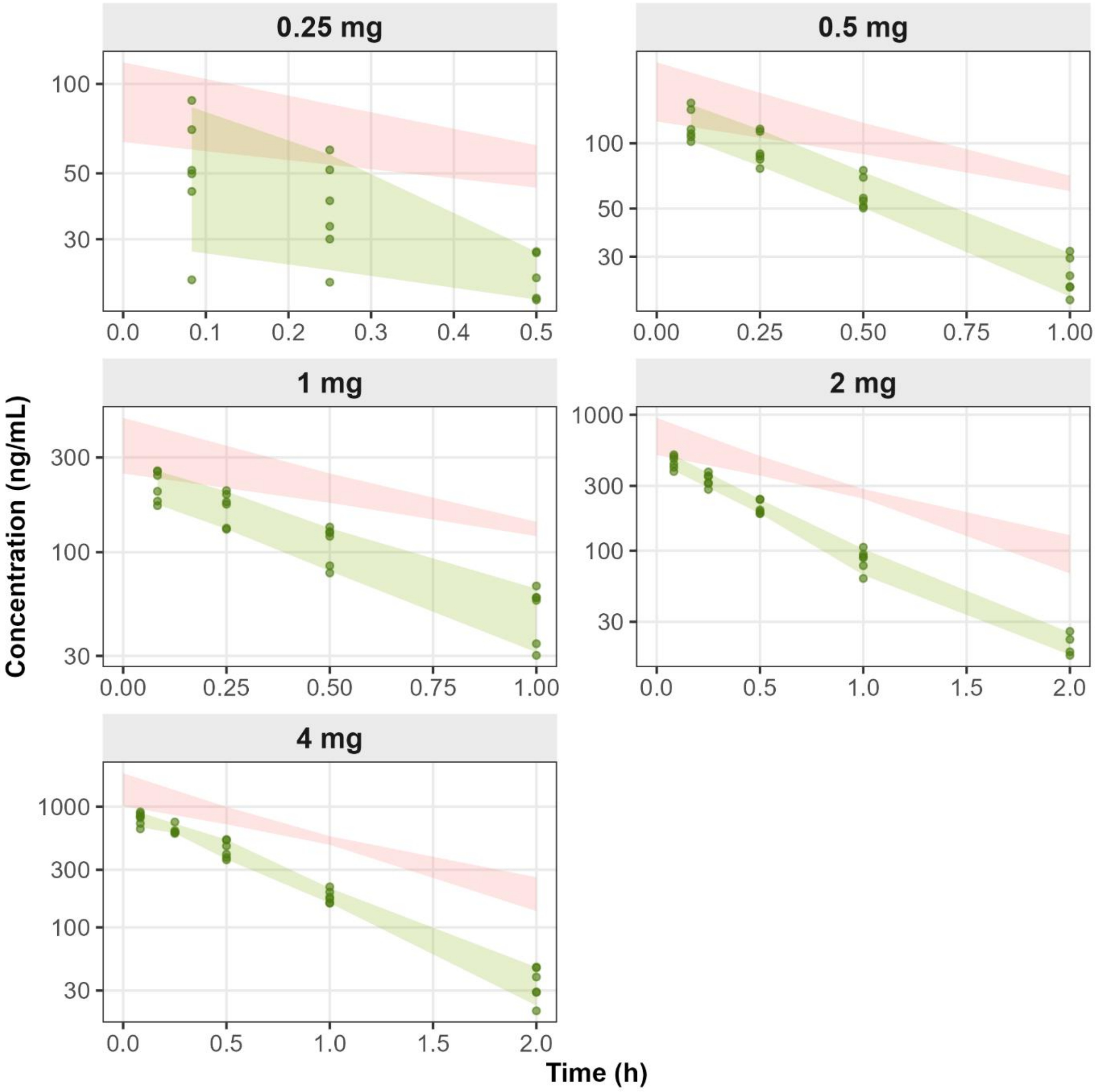

Figure S13. Comparison of NP-011 pharmacokinetic profiles between the Phase I clinical trial and simulations based on maximum life-span potential. The pink shaded area represents the 5th-95th percentile simulation interval, and the green shaded area represents the observed 5th-95th percentile interval.

Table S1. Design and sampling characteristics of the preclinical pharmacokinetic studies

Species	Dose (mg/kg)	Number of animals		Age	Body weight	Dose regimen	PK sampling
		Male	Female				
Mice	0.0214	30	0	6-10 weeks	23.60-38.12 g	Single IV bolus	Predose, 0.083, 0.25, 0.5, 1, 2, 4, 6, 8, 24 h
	0.0854	30	30				Same as above
	0.342	30	0				Same as above
Rats	0.00798	3	0	6-9 weeks	228.3-368.3 g	Single IV bolus	Predose, 0.083, 0.25, 0.5, 1, 2, 4, 6, 8, 24 h
	0.0426	3	3				Same as above
	0.171	3	0				Same as above
Monkeys	0.00534	3	0	6-10 months	2.55-4.75 kg	Single IV bolus	Predose, 0.083, 0.5, 1, 2, 4, 6, 8, 24 h
	0.0213	3	3			IV bolus, once daily for 14 days	Intensive sampling on Days 1 and 14; predose on Days 3–13
	0.0854	3	0			Single IV bolus	Predose, 0.083, 0.5, 1, 2, 4, 6, 8, 24 h

Notes: In mice, cross-sectional sampling was performed, with three animals sampled at each nominal time point; thus, each animal contributed one plasma concentration observation. In rats and monkeys, serial blood sampling was performed in the same animals. BLQ concentrations at predose and before T_{max} were set to zero, whereas BLQ concentrations after T_{max} were excluded from the NCA. Among nominal post-dose samples, 44, 43, and 31 BLQ observations were identified in the mouse, rat, and monkey datasets, respectively. One anomalous rat concentration observation was excluded from PK parameter calculation.

Table S2: Phase I study design, participant allocation, and PK sampling scheme.

Notes: All NP-011-treated participants were included in the clinical PK population. In the MAD part, one NP-011-treated participant and one placebo-treated participant

Study part	Cohort	NP-011 dose	NP-011 dosed, n	Placebo dosed, n	Body weight of NP-011-treated participants, kg, mean ± SD	Dosing regimen
SAD	1A	0.25 mg	6	2	73.05 ± 19.02	Single IV bolus dose on Day 1
	1B	0.5 mg	6	2	79.50 ± 9.00	
	1C	1 mg	6	2	74.48 ± 17.25	
	1D	2 mg	6	2	69.73 ± 8.52	
	1E	4 mg	6	2	67.30 ± 10.11	
SAD total	-	-	30	10	72.81 ± 13.26	-
MAD	2A	1 mg	7	2	72.14 ± 8.62	IV bolus once daily on Days 1-7
	2B	2 mg	6	3	72.25 ± 11.10	
	2C	4 mg	6	2	75.37 ± 15.80	
MAD total	-	-	19	7	73.19 ± 11.43	-

discontinued after the first dose because of poor venous access. The assay LLOQ was 15.62 ng/mL. In the clinical NCA, BLQ concentrations before T_{max} were set to zero, whereas those after T_{max} were treated as missing. SAD PK sampling was performed predose and at 0.083, 0.25, 0.5, 1, 2, 4, 8, 12, 24, and 48 h after dosing.

Table S3. PK parameters of NP-011 in mice through NCA.

PK parameters	Dose (mg/kg)							
	0.0214		0.0854 (male)		0.0854 (female)		0.342	
	Mean	SD	Mean	SD	Mean	SD	Mean	SD
C_{max} (µg/L)	410	92.3	1668	114	1654	330	9212	2946
Dose-normalized C_{max}	19.1	4.31	19.5	1.33	19.4	3.87	26.9	8.62
AUC_{last} (µg/L*h)	64.3	12.7	287	39.5	195	46	1352	314
Dose-normalized AUC_{last}	3.01	0.591	3.36	0.462	2.28	0.539	3.95	0.917
AUC_{inf} (µg/L*h)	64.7	12.8	287	39.4	195	46	1353	313
Dose-normalized AUC_{inf}	3.02	0.599	3.37	0.462	2.28	0.538	3.96	0.916
Half-life (h)	0.135	0.0152	0.215	0.0259	0.244	0.0557	1.21	0.904
V_d (L/kg)	0.0655	0.0097	0.0946	0.0257	0.167	0.0844	0.441	0.303
CL (L/h/kg)	0.34	0.069	0.301	0.0445	0.457	0.122	0.263	0.0664
AUC: area under the curve; V_d: volume of distribution; CL: clearance; SD: standard deviation.								

Table S4. PK parameters of NP-011 in rats through NCA.

PK parameters	Dose (mg/kg)							
	0.00798		0.0426 (male)		0.0426 (female)		0.171	
	Mean	SD	Mean	SD	Mean	SD	Mean	SD
C_{max} (µg/L)	155	55	693	265	995	613	1449	316
Dose-normalized C_{max}	19.4	6.89	16.3	6.21	23.4	14.4	8.48	1.85
AUC_{last} (µg/L*h)	18.7	4.21	62.8	15.6	78.8	42.2	270	79.9
Dose-normalized AUC_{last}	2.35	0.528	1.47	0.367	1.85	0.992	1.58	0.467
AUC_{inf} (µg/L*h)	18.8	4.22	63	15.5	78.9	42.2	271	81
Dose-normalized AUC_{inf}	2.35	0.529	1.48	0.365	1.85	0.992	1.58	0.474
Half-life (h)	0.127	0.00729	1.08	0.858	0.175	0.0446	1.16	1.12
V_d (L/kg)	0.0814	0.0221	1.19	0.964	0.183	0.144	0.934	0.673
CL (L/h/kg)	0.442	0.114	0.702	0.156	0.709	0.489	0.672	0.205

AUC: area under the curve; V_d: volume of distribution; CL: clearance; SD: standard deviation.

Table S5. PK parameters of NP-011 in monkeys through NCA.

PK parameters	Dose (mg/kg)											
	Day 1			Day 1			Day 14			Day 1		
	0.00534		0.0213 (male)		0.0213 (female)		0.0213 (male)		0.0213 (female)		0.0854	
	Mean	SD	Mean	SD	Mean	SD	Mean	SD	Mean	SD	Mean	SD
C_{max} (μg/L)	126	22.1	444	22.5	408	69.3	477	97.4	311	272	2055	450
Dose-normalized C_{max}	23.5	4.13	20.9	1.05	19.2	3.25	22.4	4.57	14.6	12.8	24.1	5.27
AUC_{last} (μg/L*h)	60.7	2.44	203	14.9	214	40.5	253	39.1	215	7.29	1165	86.4
Dose-normalized AUC_{last}	11.4	0.457	9.54	0.7	10	1.9	11.9	1.84	10.1	0.342	13.6	1.01
AUC_{inf} (μg/L*h)	61	2.52	203	15	214	40.2	254	39.3	216	7.48	1166	86.2
Dose-normalized AUC_{inf}	11.4	0.471	9.55	0.703	10	1.89	11.9	1.84	10.2	0.351	13.7	1.01
Half-life (h)	0.368	0.0722	0.452	0.11	1.76	1.95	1.91	0.498	4.08	4.02	0.648	0.0411
V_d (L/kg)	0.0467	0.00995	0.0678	0.0136	0.299	0.375	0.229	0.0265	0.577	0.562	0.0689	0.00921
CL (L/h/kg)	0.0877	0.0037	0.105	0.00768	0.102	0.0215	0.0852	0.0124	0.0985	0.00346	0.0735	0.00534

AUC: area under the curve; *V*d: volume of distribution; CL: clearance; SD: standard deviation.

Table S6. Specification and estimation details of the final population pharmacokinetic models.

Model Feature	Mice	Rats	Monkeys
Model structure			
Structural model	Two-compartment	Two-compartment	Two-compartment
Administration; animals (n)	IV bolus; 120 (cross-sectional sampling)	IV bolus; 12	IV bolus; 12
NONMEM implementation	ADVAN6/TRANS1 (TOL 9)	ADVAN6/TRANS1 (TOL 9)	ADVAN6/TRANS1 (TOL 9)
Estimation method	FOCE with interaction	FOCE with interaction	FOCE with interaction
IIV parameters	CL, V_P	CL	CL, V_C
Ω structure	Diagonal	Diagonal	Diagonal
Residual error	Proportional (log scale)	Proportional (log scale)	Proportional (log scale)

Notes: Initial parameter values in the final control streams were set to the converged estimates obtained from preceding model-development runs. Minimization and covariance steps were completed successfully for all final models.

Table S7. Bootstrap performance and shrinkage diagnostics of the final population PK models.

Metric / Parameter	Mice	Rats	Monkeys
Bootstrap performance (1,000 individual-level resamples)			
Successful bootstrap runs, n (%)	981 (98.1)	997 (99.7)	996 (99.6)
Terminated due to rounding errors, n (%)	19 (1.9)	3 (0.3)	4 (0.4)
Estimates near a boundary, n (%)	377 (37.7)	324 (32.4)	467 (46.7)
Shrinkage diagnostics (%)			
ETA shrinkage, CL	7.9	31.6	50.4
ETA shrinkage, V_C	-	-	7.8
ETA shrinkage, V_P	43.9	-	-
EPS shrinkage	10.6	4.1	4.3

Notes: Bootstrap analyses were based on 1,000 individual-level resamples. Parameter estimates and corresponding nonparametric 95% confidence intervals are reported in Table 1 of the main manuscript. ETA, inter-individual random effect; EPS, residual random effect.

Table S8. Modification of simple allometry.

Condition	Modified equation	Method
$b < 0.7$	Simple allometry, $Y = axBW^b$	SA
$0.7 \leq b < 1$	ROE with Maximum life-span potential $Y * MLP = axBW^b$	MLP
$b \geq 1$	ROE with Brain weight $Y * BrW = axBW^b$	BrW

Table S9. Brain weight of species [1].

Species	Mouse	Rat	Monkey	Human
Brain weight (% body weight)	1.65	0.57	1.56	2

Table S10. Allometric regression and sensitivity analyses for human pharmacokinetic extrapolation.

Panel A. Allometric regression of total pharmacokinetic parameters against body weight

Parameter	Coefficient *a*	Exponent *b*	SE (*b*)	95% CI (*b*)	R^2	Human (70 kg)
CL (L/h)	0.192	0.703	0.267	- 2.69 to 4.10	0.874	3.80
V_C (L)	0.059	0.914	0.081	- 0.12 to 1.94	0.992	2.88
Q (L/h)	0.006	0.867	0.323	- 3.24 to 4.97	0.878	0.25
V_P (L)	0.072	1.374	0.101	0.09 to 2.66	0.995	24.61

Panel B. Leave-one-species-out sensitivity analysis of allometric exponents

Parameter	Full model (3 species)	Mice excluded	Rats excluded	Monkeys excluded
CL (L/h)	0.703	0.254	0.707	1.181
V_C (L)	0.914	0.778	0.915	1.059
Q (L/h)	0.867	0.325	0.871	1.444
V_P (L)	1.374	1.205	1.375	1.555

Panel C. Fixed-effects sensitivity analysis compared with variability-based simulation medians

Dose (mg)	AUC_{inf}, IIV median	AUC_{inf}, fixed effects	Difference (%)	C_{max}, IIV median	C_{max}, fixed effects	Difference (%)
0.25	65.8	65.9	- 0.1	86.6	86.8	- 0.3
0.5	131.9	131.7	+ 0.1	173.3	173.6	- 0.2
1	263.7	263.4	+ 0.1	345.0	347.2	- 0.6
2	526.2	526.9	- 0.1	694.6	694.3	+ 0.0
4	1053.4	1053.8	- 0.0	1391.1	1388.6	+ 0.2

Notes: Leave-one-species-out analyses sequentially excluded each species. Fixed-effects predictions used the typical human structural parameters without inter-individual variability; $t_{1/2,\alpha}$ and $t_{1/2,\beta}$ were approximately 0.49 and 72.7 h, respectively, under both approaches. The maximum absolute difference was 0.63% based on unrounded values.

Table S11. Coverage of observed Phase I concentrations within the simulated 5th–95th percentile prediction intervals under the SA and MLP methods.

Dose (mg)	Total observations, n	SA: within interval, n (%)	MLP: within interval, n (%)
0.25	17	4 (23.5)	3 (17.6)
0.5	24	4 (16.7)	4 (16.7)
1	24	6 (25.0)	3 (12.5)
2	28	5 (17.9)	2 (7.1)
4	30	2 (6.7)	0 (0.0)
Overall	123	21 (17.1)	12 (9.8)

Coverage is the number and proportion of observed concentrations falling within the simulated interval at each dose level and overall.

Table S12. Human-predicted PK parameters of NP-011 using a maximum life-span potential .

Dose (mg)	Median AUC_{inf} (ng/mL*h)	90% prediction interval AUC_{inf} (ng/mL*h)	Median C_{max} (ng/mL)	90% prediction interval for C_{max} (ng/mL)	Model-derived $t_{1/2,\alpha}$ (h), median (90% PI)	Model-derived $t_{1/2,\beta}$ (h), median (90% PI)
0.25	97	90 - 105	87	64 - 118	0.728 (0.528-1.001)	107.34 (106.86-107.87)
0.5	194	180 – 210	173	126 - 236	0.729 (0.527-1.011)	107.36 (106.86-107.89)
1	389	360 - 420	345	249 - 475	0.734 (0.526-1.018)	107.36 (106.86-107.90)
2	775	720 – 838	695	507 - 949	0.725 (0.528-1.006)	107.34 (106.87-107.89)
4	1552	1439 - 1679	1391	1011 - 1886	0.726 (0.530-1.004)	107.35 (106.86-107.90)

AUC, area under the plasma concentration–time curve; C_{max}, maximum plasma concentration; $t_{1/2,\alpha}$, model-derived distribution half-life; $t_{1/2,\beta}$, model-derived terminal half-life; PI, prediction interval. The hybrid half-lives were calculated for each simulation replicate from the corresponding CL, V_C, Q, and V_P values.

Table S13. Comparison of observed and MLP-corrected predicted Phase I exposure metrics by dose level.

Dose (mg)	Observed AUC_{inf}, median (n)	P/O ratio for AUC_{inf}	%PE for AUC_{inf}	Observed C_{max} median (ng/mL)	P/O ratio for C_{max}	%PE for C_{max}
0.25	34 (5)	2.89	189	61	1.42	42
0.5	74 (6)	2.62	162	130	1.33	33
1	163 (6)	2.38	138	259	1.33	33
2	286 (6)	2.71	171	505	1.38	38
4	595 (6)	2.61	161	940	1.48	48

AUC_{inf}, area under the plasma concentration–time curve extrapolated to infinity; C_{max}, maximum plasma concentration; P/O, predicted-to-observed ratio; %PE, percentage prediction error. Comparisons were performed using dose-group medians.

1. Mahmood, I. Application of Allometric Principles for the Prediction of Pharmacokinetics in Human and Veterinary Drug Development. *Advanced Drug Delivery Reviews* **2007**, *59*, 1177–1192, doi:10.1016/j.addr.2007.05.015.
